# Titanium-Doped Diamond-like Carbon Layers as a Promising Coating for Joint Replacements Supporting Osteogenic Differentiation of Mesenchymal Stem Cells

**DOI:** 10.3390/ijms25052837

**Published:** 2024-02-29

**Authors:** Martina Travnickova, Elena Filova, Petr Slepicka, Nikola Slepickova Kasalkova, Tomas Kocourek, Margit Zaloudkova, Tomas Suchy, Lucie Bacakova

**Affiliations:** 1 Laboratory of Biomaterials and Tissue Engineering, Institute of Physiology of the Czech Academy of Sciences, Videnska 1083, 142 00 Prague, Czech Republic; martina.travnickova@fgu.cas.cz (M.T.); lucie.bacakova@fgu.cas.cz (L.B.); 2 Faculty of Materials and Technology, VSB-Technical University of Ostrava, 17. Listopadu 2172/15, 708 00 Ostrava, Czech Republic; 3 Department of Solid State Engineering, University of Chemistry and Technology Prague, Technicka 5, 166 28 Prague, Czech Republic; petr.slepicka@vscht.cz (P.S.); nikola.kasalkova@vscht.cz (N.S.K.); 4 Institute of Physics of the Czech Academy of Sciences, Na Slovance 2, 182 21 Prague, Czech Republic; kocourek@fzu.cz; 5 Faculty of Biomedical Engineering, Czech Technical University in Prague, Nam. Sitna 3105, 272 01 Kladno, Czech Republic; 6 Institute of Rock Structure and Mechanics, Czech Academy of Sciences, V Holesovickach 94/41, 182 09 Prague, Czech Republic; zaloudkova@irsm.cas.cz (M.Z.); suchyt@irsm.cas.cz (T.S.)

**Keywords:** titanium, diamond-like carbon layer (DLC), biocompatibility, osteogenic differentiation, adipose tissue-derived stem cells (ADSCs), bone marrow mesenchymal stem cells (bmMSCs)

## Abstract

Diamond-like carbon (DLC) layers are known for their high corrosion and wear resistance, low friction, and high biocompatibility. However, it is often necessary to dope DLC layers with additional chemical elements to strengthen their adhesion to the substrate. Ti-DLC layers (doped with 0.4, 2.1, 3.7, 6.6, and 12.8 at.% of Ti) were prepared by dual pulsed laser deposition, and pure DLC, glass, and polystyrene (PS) were used as controls. In vitro cell–material interactions were investigated with an emphasis on cell adhesion, proliferation, and osteogenic differentiation. We observed slightly increasing roughness and contact angle and decreasing surface free energy on Ti-DLC layers with increasing Ti content. Three-week biological experiments were performed using adipose tissue-derived stem cells (ADSCs) and bone marrow mesenchymal stem cells (bmMSCs) in vitro. The cell proliferation activity was similar or slightly higher on the Ti-doped materials than on glass and PS. Osteogenic cell differentiation on all materials was proved by collagen and osteocalcin production, ALP activity, and Ca deposition. The bmMSCs exhibited greater initial proliferation potential and an earlier onset of osteogenic differentiation than the ADSCs. The ADSCs showed a slightly higher formation of focal adhesions, higher metabolic activity, and Ca deposition with increasing Ti content.

## 1. Introduction

Discovering new bioinert or bioactive coatings for conventionally used joint implants, made from cobalt (Co)–chromium (Cr) alloys, magnesium (Mg) alloys, stainless steel, or titanium (Ti) alloys, is still of high importance. For example, new coatings can provide improved chemical stability, strengthened corrosion resistance, appropriate physiological mechanical properties, better support for osseointegration, or increased microbial resistance for current orthopaedic implants (for a review, see [1,2,3,4,5]). One of the most important properties of joint replacements is high wear resistance, which can be obtained by creating a stable surface layer on the material. The stable surface layer plays a key role in preventing the release of metal ions and wear debris from an implant, which can otherwise later result in inflammation of the surrounding tissue followed by osteolysis and implant failure/loosening (for a review, see [6,7]).

Diamond-like carbon (DLC) layers have been used for coating orthopedic implants (e.g., joint replacements, medical wires, screws) due to their hardness, high corrosion resistance, low friction, high wear resistance, and high biocompatibility (for a review, see [8,9,10]). Moreover, due to their chemical inertness, surface smoothness, and hydrophobicity, resulting in low thrombogenicity, DLC coatings have been widely explored for cardiovascular stents, heart valve prostheses, and other cardiovascular devices (for a review, see [8,9,10]). Nevertheless, the DLC layer itself is burdened with instability in the aqueous environment and poor substrate adhesion [11]. These disadvantages can be overcome by using suitable interlayers or by doping DLC with specific chemical elements during fabrication (for a review, see [8,10]). Ti is known to possess superior biocompatibility properties, and it is therefore a commonly used material for dental implants and medical devices (for a review, see [12,13]). The addition of Ti or Cr to DLC has been reported to increase the adhesion of DLC layers to an underlying substrate, such as Si(100) wafers, 316 L stainless steel, or Ti6Al4V [14,15,16,17,18,19,20]. The addition of Ti to DLC also improves other mechanical properties, such as the elastic modulus, hardness, and surface roughness of the coating [15,16,21]. Similarly, our previous studies on the fabrication of Ti-DLC layers showed an increasing critical load and increasing roughness as the Ti content increases, along with the Young’s modulus of the Ti-DLC being closer to that of the cortical bone, in comparison with the results when pure DLC layers are used [16]. The reported properties depended on the specific Ti content and fabrication techniques, such as dual pulsed laser deposition (dual PLD) or pulsed laser deposition combined with magnetron sputtering (PLD + MS) [16,21]. In addition to the improved physicochemical properties, the high initial biocompatibility of Ti-DLC layers doped with various contents of Ti was reported in our previous studies [16,21] and also by other researchers [15].

Mesenchymal stem cells (MSCs), which are able to undergo osteogenic and/or chondrogenic differentiation, play a key role in bone regeneration and bone healing/repair processes (for a review, see [22]). Under physiological conditions in vivo, MSCs are recruited to the damaged area from the surrounding tissue (mainly from the periosteum, the endosteum, and the bone marrow cavity) as well as from peripheral circulation [22]. The MSCs involved in bone repair are modulated by many signaling pathways, which can be further advantageously used in regenerative medicine to stimulate the mobilization and homing of the MSCs (for a review, see [23]). For decades, bone marrow mesenchymal stem cells (bmMSCs) have been widely investigated, and these cells have been considered as a gold standard for bone cell-based therapies. However, MSCs can also be harvested from other specific tissues such as adipose tissue (ADSCs), dental pulp, peripheral blood, amniotic fluid, or the placenta (for a review, see [24]). Among these cells/tissues, ADSCs are one of the most promising cell types for MSC-based bone regeneration therapies due to their easy accessibility in relatively large quantities, high proliferative capacity, high osteogenic differentiation potential, osteoinductive and immunomodulation properties (for a review, see [25]). These superior characteristics rank ADSCs equal to or even higher than bmMSCs for further routine autologous or allogenic clinical applications in bone tissue engineering. However, previous and current clinical trials focused on the usage of ADSCs in bone repair have so far resulted in ambiguous outcomes, and specific cell characterization therefore needs to be performed to allow for comparisons of the results/outcomes that are obtained (for a review, see [26]). To the best of our knowledge, no comprehensive study covering the interaction between variably Ti-doped DLC coatings and adult MSCs has been performed. Similar Ti-DLC coatings have been fabricated and characterized in the past, and their basic cytocompatibility has been tested with the Saos-2 cell line, i.e., human osteoblast-like cells derived from osteosarcoma, by our group [16,21].

The aim of the current study was to analyze in vitro the adhesion, growth, and differentiation of primary or low-passaged human osteogenic cells, namely, ADSCs and bmMSCs on Ti-DLC layers, and thus to ensure the suitability of these layers for coating orthopedic implants. Another aim was to elucidate the potential influence of an increasing Ti content on the behavior of adult MSCs.

For this purpose, multipotent MSCs that are physiologically involved in bone healing or that are present in a surrounding tissue were chosen. Specifically, cell-material interactions of two types of adult MSCs (i.e., ADSCs and bmMSCs) were studied. Detailed physicochemical characterization of the prepared Ti-DLC layers was performed prior to the cell experiments.

## 2. Results

### 2.1. Material Characterization

#### 2.1.1. Surface Morphology

**Figure 1:**
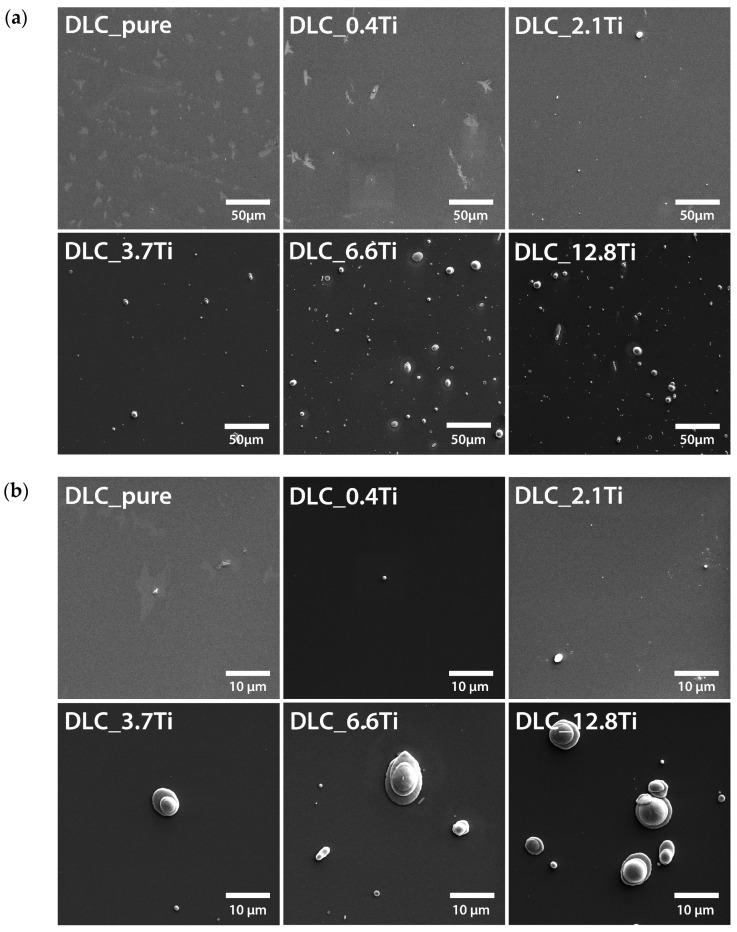
SEM images of pure DLC and DLC doped with Ti (0.4, 2.1, 3.7, 6.6, and 12.8 at.% of Ti). Scale bar = 50 µm (**a**) and 10 µm (**b**).

The surface morphology of the samples was determined by scanning electron microscope (SEM) and atomic force microscopy (AFM) techniques. As shown in the SEM images (Figure 1), with increasing amounts of Ti in the DLC layers, larger clusters were formed on the surface. These clusters were probably formed during the PLD process on the pure DLC substrate. The structures were relatively randomly distributed over the surface, with globular shapes and diameters of several micrometers. As expected, the size, number, and concentration of these structures increased with increasing thickness of the deposited layer.

**Figure 2:**
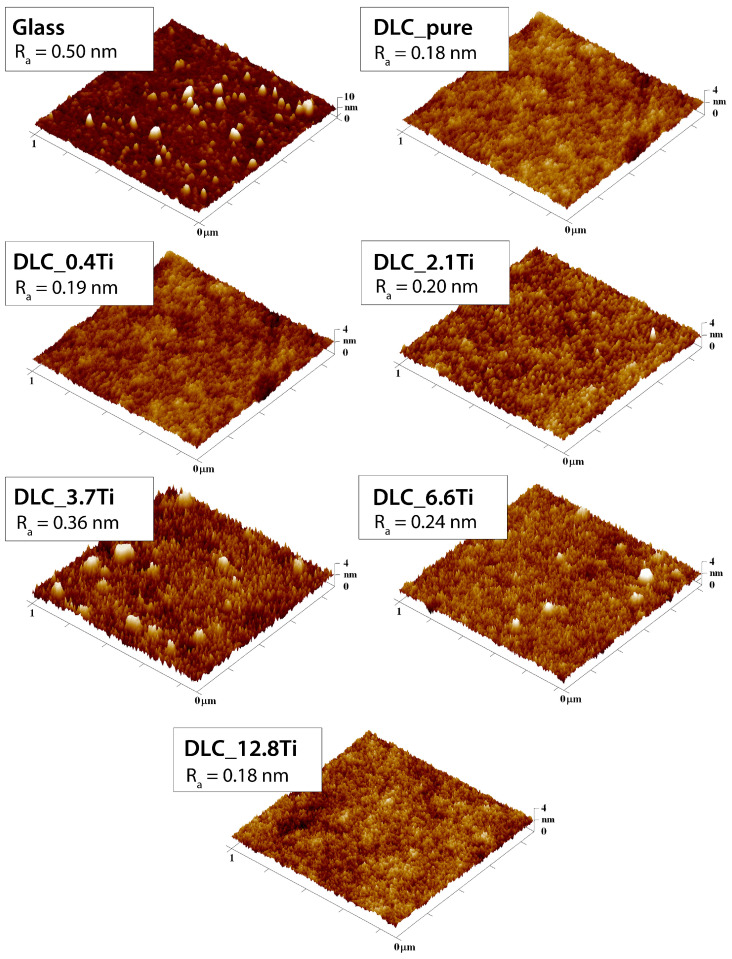
AFM scans of glass, of pure DLC and DLC doped with Ti (0.4, 2.1, 3.7, 6.6, and 12.8 at.% of Ti). A region of 1 µm × 1 µm is depicted. The mean roughness value (Ra) represents the average surface roughness in nm.

However, it is clear from the SEM images (Figure 1) and also from the AFM images (Figure 2) that most of the surface area is not significantly affected by these clusters. A detailed analysis of the surface morphology with AFM confirmed that no significant changes were found on the DLC surface with increasing amounts of titanium. It can be seen that there was a slight increase in roughness with increasing thickness of Ti, followed by a decrease in roughness for the highest concentration of Ti, i.e., 12.8 at.% of Ti. However, the changes indicated by the AFM technique were within the range of 0.1–0.4 nm, confirming good homogeneity in the prepared nanolayers. The nanostructure of the layer was not significantly affected by an increasing amount of Ti.

#### 2.1.2. Wettability

It is known that the surface wettability value depends mainly on the chemical composition of the surface layer. Other surface parameters, such as surface morphology and roughness, can also have an influence on this value. In this case, however, the “very smooth” surface roughness values of the samples are very small (tenths of nm), and their influence on the change in surface wettability can therefore be neglected. Table 1 shows the water and glycerol contact angles and the total surface free energy (SFE) values for the original glass substrate and for the substrate with a deposited DLC layer containing 0–12.8 at.% of Ti. It can be seen that the deposition of the DLC layer on the glass substrate led to a significant increase in the value of the contact angle, i.e., an increase in the hydrophobicity of the surface. The addition of Ti to the deposited layer caused a further change in the contact angle. It can be stated that as the amount of Ti in the DLC layer increases, the value of the contact angle also increases slightly.

It is known from experiments that cells adhere preferentially and better to surfaces with moderate hydrophilicity, i.e., those that are neither too wettable nor too hydrophobic. From the point of view of surface wettability, surfaces with contact angle values in the range of 70°–80° can be expected to be the most suitable for cell cultivation. These values correspond to samples with a deposited DLC layer containing a medium amount of Ti (2.1–6.6 at.%).

#### 2.1.3. Surface Free Energy (SFE)

Surface free energy determines the disruption of intermolecular bonds on the surface when the surface is created. The SFE of a solid surface is the parameter that can provide information about how liquids will interact with the solid surface. The SFE of the surface also affects its wettability. In general, it can be said that a surface with high SFE is more easily wetted than a surface with low SFE. The highest surface energy value was measured on DLC_0.4Ti and DLC_pure, and a lower SFE was observed on DLC with higher Ti concentrations, with the lowest value on DLC_12.8Ti.

#### 2.1.4. Chemical Composition

The chemical composition of the glass, the pure DLC layer, and the Ti-doped DLC layers were studied using two different techniques: X-ray photoelectron spectroscopy (XPS) and energy dispersive X-ray spectroscopy (EDS). In the case of the XPS method, two different take-off angles to the perpendicular of the sample surface were used. Table S1 (take-off angle 0°) summarizes the atomic concentrations of chemical elements on the surface of the sample measured by XPS. The Ti concentrations increased from 0.89 at.% (sample DLC_0.4Ti) to 9.45 at.% (sample DLC_12.8Ti), and the C concentrations decreased from 93.45 at.% (sample DLC_0.4Ti) to 68.41 at.% (sample DLC_12.8Ti). The O concentrations increased with increasing Ti concentrations, and the N concentrations were almost unchanged.

**Figure 3:**
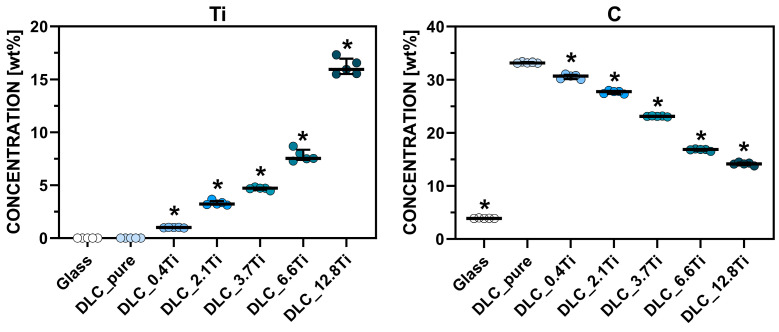
The mass concentration of Ti and C elements determined by EDS on the surface of the tested materials. The Mann–Whitney test was applied to compare each material separately with the control sample (DLC_pure). The pairs with *p*-values ≤ 0.05 (n = 5) are marked with “*”.

Figure 3 shows detailed mass concentrations of Ti and C measured by EDS. The EDS analysis of Ti-doped DLC layers proved the presence of Ti in an increasing manner, ranging from 1 wt.% (sample DLC_0.4Ti) to 15.95 wt.% (sample DLC_12.8Ti), and the presence of C in a decreasing manner, ranging from 30.71 wt.% (sample DLC_0.4Ti) to 14.15 wt.% (sample DLC_12.8Ti). All these values were significantly different from the DLC_pure sample. The chemical analyses show that Ti is distributed throughout the entire analyzed Ti-doped DLC layers.

Table S2 (take-off angle 81°) provides information on the elemental composition of the superficial surface layers. It can be seen that a large quantity of C was detected in the pure glass sample. The high values are caused by so-called surface carbonaceous contamination from the ambient atmosphere. Table S3 summarizes the concentrations of all elements determined by EDS. The EDS method can analyze the chemical composition of samples to a “greater depth” than the XPS method. In contrast to the XPS method, elements that occur in silica glasses were also detected here (in the case of the XPS method, however, the amounts of these elements were below the detection limit). It is evident that, in this analysis, the phenomenon of so-called carbon contamination from the air atmosphere is significantly less pronounced.

### 2.2. Biological In Vitro Experiments

#### 2.2.1. Cell Proliferation and Metabolic Activity

Cell proliferation on the studied materials was estimated by direct cell counting and by a resazurin metabolic assay, which is considered to be an indirect indicator of cell growth. In general, the ADSCs and bmMSCs showed a very high biocompatibility rate of the Ti-doped DLC layers, which was proved by very similar or even higher proliferation than in the case of the control PS material.

**Figure 4:**
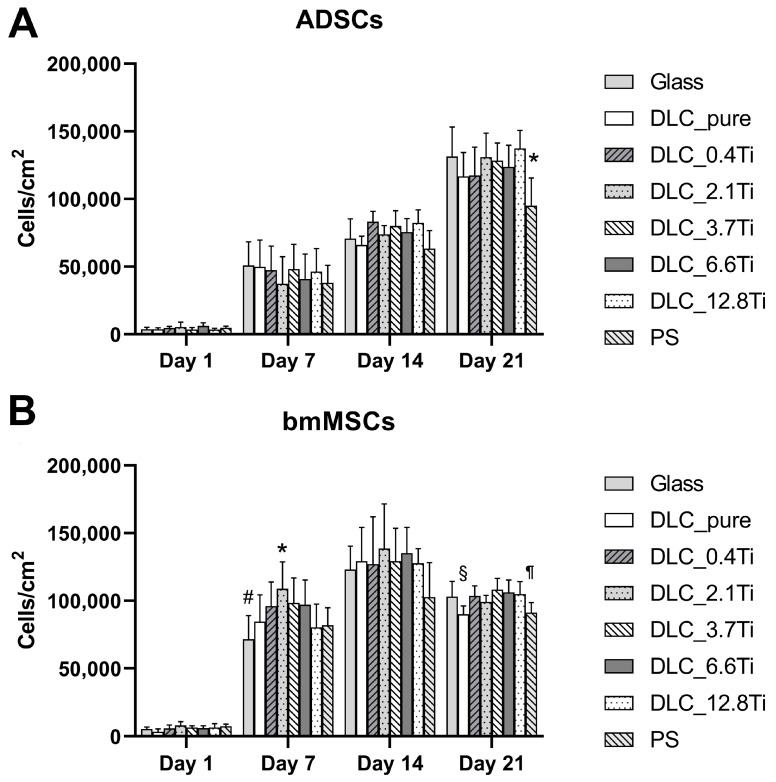
The cell population densities of ADSCs (**A**) and bmMSCs (**B**) on glass, on pure DLC, on DLC doped with Ti (0.4, 2.1, 3.7, 6.6, and 12.8 at.% of Ti), and on polystyrene (PS) on days 1, 7, 14, and 21. The data are expressed as mean + SD. ANOVA, Student–Newman–Keuls method. The statistical comparison among the samples was made on cells on the same day of the culture. The statistical significance (*p* ≤ 0.001) is specified above the columns (ADSCs (**A**): * means a lower value than on glass, 2.1 and 12.8 at.%, bmMSCs (**B**): * means a higher value than on glass, 0.4 and 12.8 at.%, and on PS; # means a lower value than on 0.4, 2.1, 3.7 and 6.6 at.%; § means a lower value than on 3.7 and 6.6 at.%; ¶ means a lower value than on 3.7 at.%).

**Figure 5:**
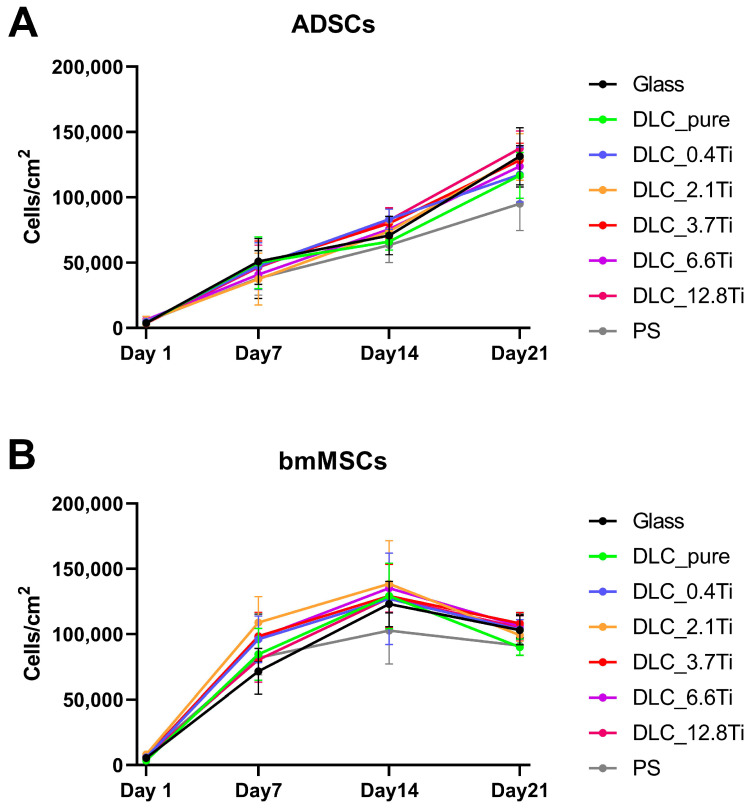
Growth curves of ADSCs (**A**) and bmMSCs (**B**) on glass, on pure DLC, on DLC doped with Ti (0.4, 2.1, 3.7, 6.6, and 12.8 at.% of Ti), and on polystyrene (PS) on days 1, 7, 14, and 21. The data are expressed as mean + SD.

**Figure 6:**
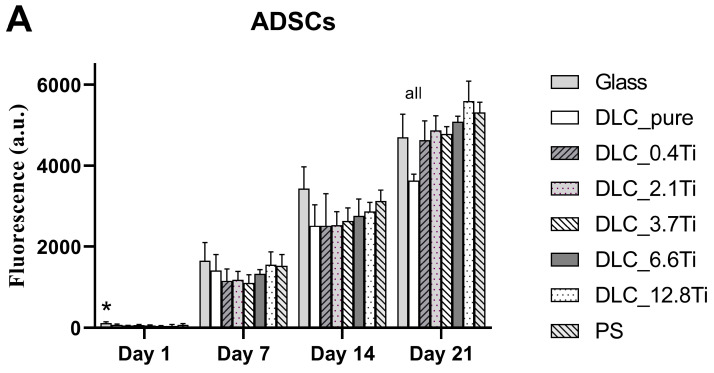

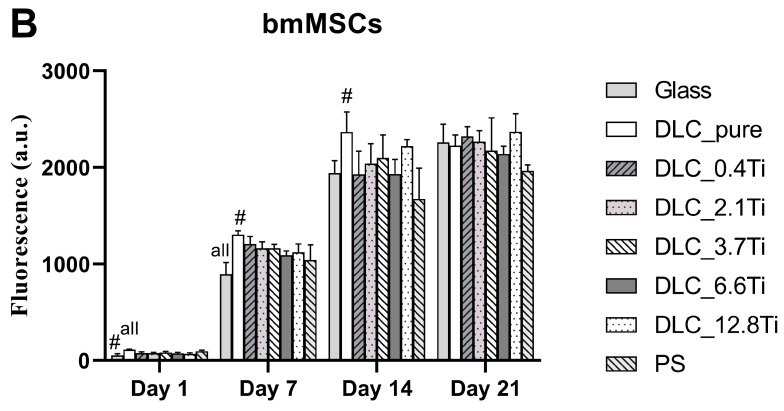
The metabolic activity of ADSCs (**A**) and bmMSCs (**B**) on glass, on pure DLC, on DLC doped with Ti (0.4, 2.1, 3.7, 6.6, and 12.8 at.% of Ti), and on polystyrene (PS) on days 1, 7, 14, and 21. The data (n = 3) are expressed as mean + SD. ANOVA, Student–Newman–Keuls method. The statistical comparison among the samples was made on cells on the same day of the culture. The statistical significance (*p* < 0.05) is specified above the columns (ADSCs (**A**): * means a higher value than on 6.6 and 12.8 at.%; “all” means a statistically different value from all other samples on the specific day of the culture, bmMSCs (**B**): # means a significantly different value compared to PS, “all” means a statistically different value from all other samples on the specific day of the culture).

The cell number of ADSCs increased in time and reached a maximum on day 21 (the end of the cell culture experiment) (Figure 4 and Figure 5). A similar tendency was also observed in the case of the metabolic activity of ADSCs (Figure 6). Only mild differences in cell number or metabolic activity were observed among the studied samples on specific days of the culture. Interestingly, in the case of the DLC pure samples, the lowest value of the metabolic activity on day 21 (Figure 6) was probably caused by the visually observable partial detachment of the surface DLC layer from the underlying glass material.

In comparison with the ADSCs, the bmMSCs manifested earlier rapid growth with maximum values on day 14 (Figure 4 and Figure 5). In the cell culture, the bmMSCs tended to form multilayers on all studied materials, which could probably have influenced the manual cell counting on days 14 and 21. Nevertheless, the metabolic activity of bmMSCs showed a similar tendency and scarcely increased between days 14 and 21 (Figure 6).

#### 2.2.2. Cell Spreading Area and Cell Morphology

**Figure 7:**
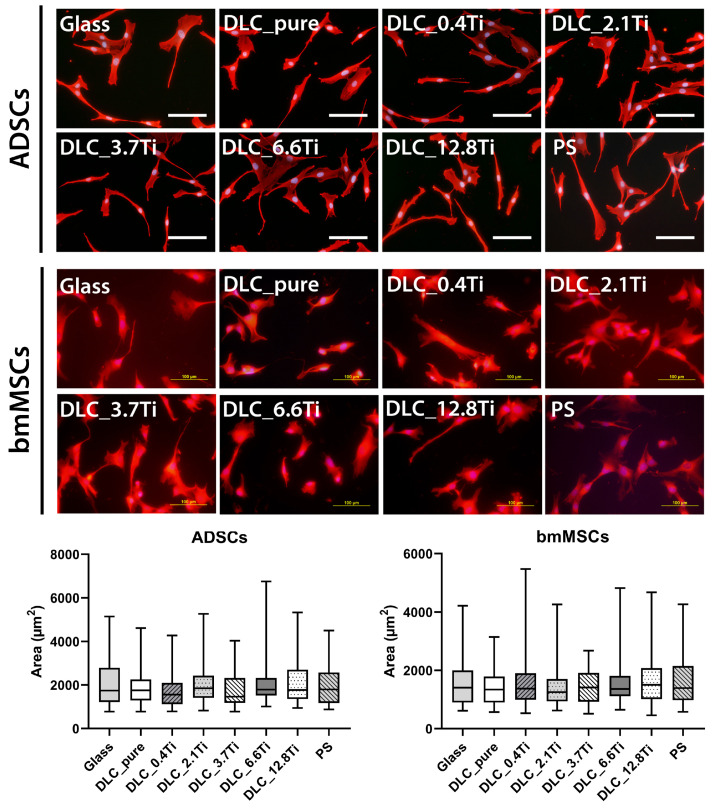
The morphology and cell spreading of ADSCs and bmMSCs on day 1 after seeding on glass, on pure DLC, on DLC doped with Ti (0.4, 2.1, 3.7, 6.6, and 12.8 at.% of Ti), and on polystyrene (PS). The cell cytoplasm was visualized by Texas Red C_2_-maleimide (red color), and the cell nuclei were counterstained with Hoechst 33258 (blue color). Representative images were selected. IX71 Olympus microscope, DP71 digital camera, objective ×20, scale bar = 100 μm. The cell spreading area was analyzed by TESCAN Atlas software, version 2.9.5.2. Data are presented as box plots with a median line, the outer edges representing the 1st and 3rd quartile, and the whiskers depicting the 5th and the 95th percentile.

The initial cell adhesion to the tested materials was quantified by measuring the cell spreading area on day 1 after seeding. The ADSCs showed a spindle-shaped morphology on all tested materials, and the cell spreading area on the DLC-based samples appeared to be similar as in the case of glass and the PS control material (Figure 7). The bmMSCs showed a polygonal-shaped morphology on both the DLC-based materials and the control materials, and no statistical differences were observed in the cell spreading area on day 1 (Figure 7).

**Figure 8:**
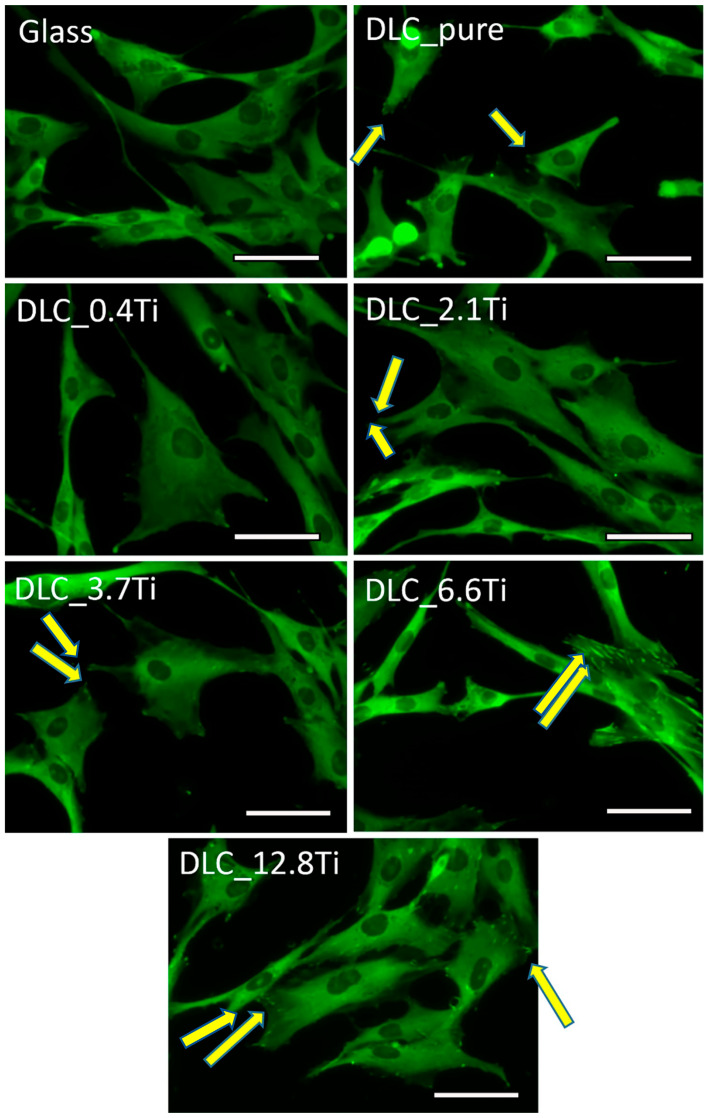
The immunofluorescence staining of vinculin in ADSCs on glass, on pure DLC, and on DLC doped with Ti (0.4, 2.1, 3.7, 6.6, and 12.8 at.% of Ti) on day 3. Representative images were selected. Olympus IX71 microscope, IX71 digital camera, objective ×20, scale bar = 50 µm. The arrows show selected focal adhesions.

On day 3, the morphology, cytoskeleton, and focal adhesions of the ADSCs were visualized. The cells were positively stained for F-actin, and these actin stress fibers showed a well-developed cytoskeleton in cells on all samples (Figure 8 and Figure S1). Vinculin staining revealed the presence of focal adhesions (i.e., plasma membrane-associated assemblies of stress fibers with cell adhesion receptors binding the extracellular matrix), which were more pronounced in cells cultured on DLC with a higher Ti content, namely, on DLC_6.6Ti and DLC_12.8Ti (Figure 8).

#### 2.2.3. Immunofluorescence Staining of Osteogenic Markers

**Figure 9:**
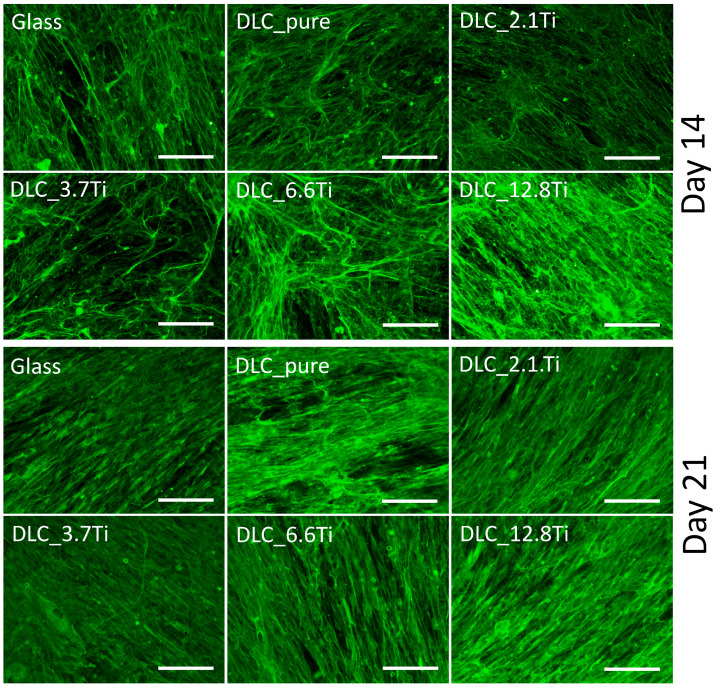
The immunofluorescence staining of type I collagen in ADSCs on glass, on pure DLC, and on DLC doped with Ti (2.1, 3.7, 6.6, and 12.8 at.% of Ti) on day 14. Representative images were selected. Olympus IX71 microscope, IX71 digital camera, objective ×20, scale bar = 100 µm.

**Figure 10:**
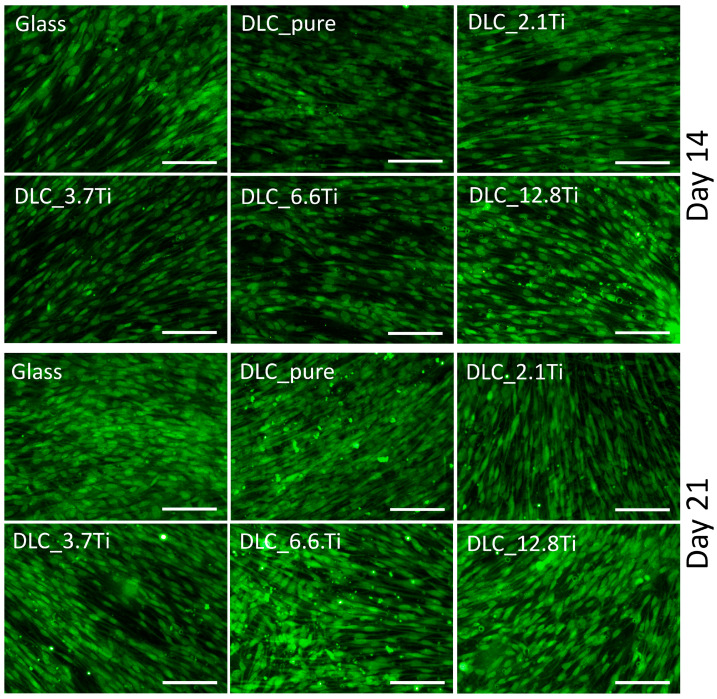
The immunofluorescence staining of osteocalcin in ADSCs on glass, on pure DLC, and on DLC doped with Ti (2.1, 3.7, 6.6, and 12.8 at.% of Ti) on days 14 and 21. Representative images were selected. Olympus IX71 microscope, IX71 digital camera, objective ×20, scale bar = 100 µm.

**Figure 11:**
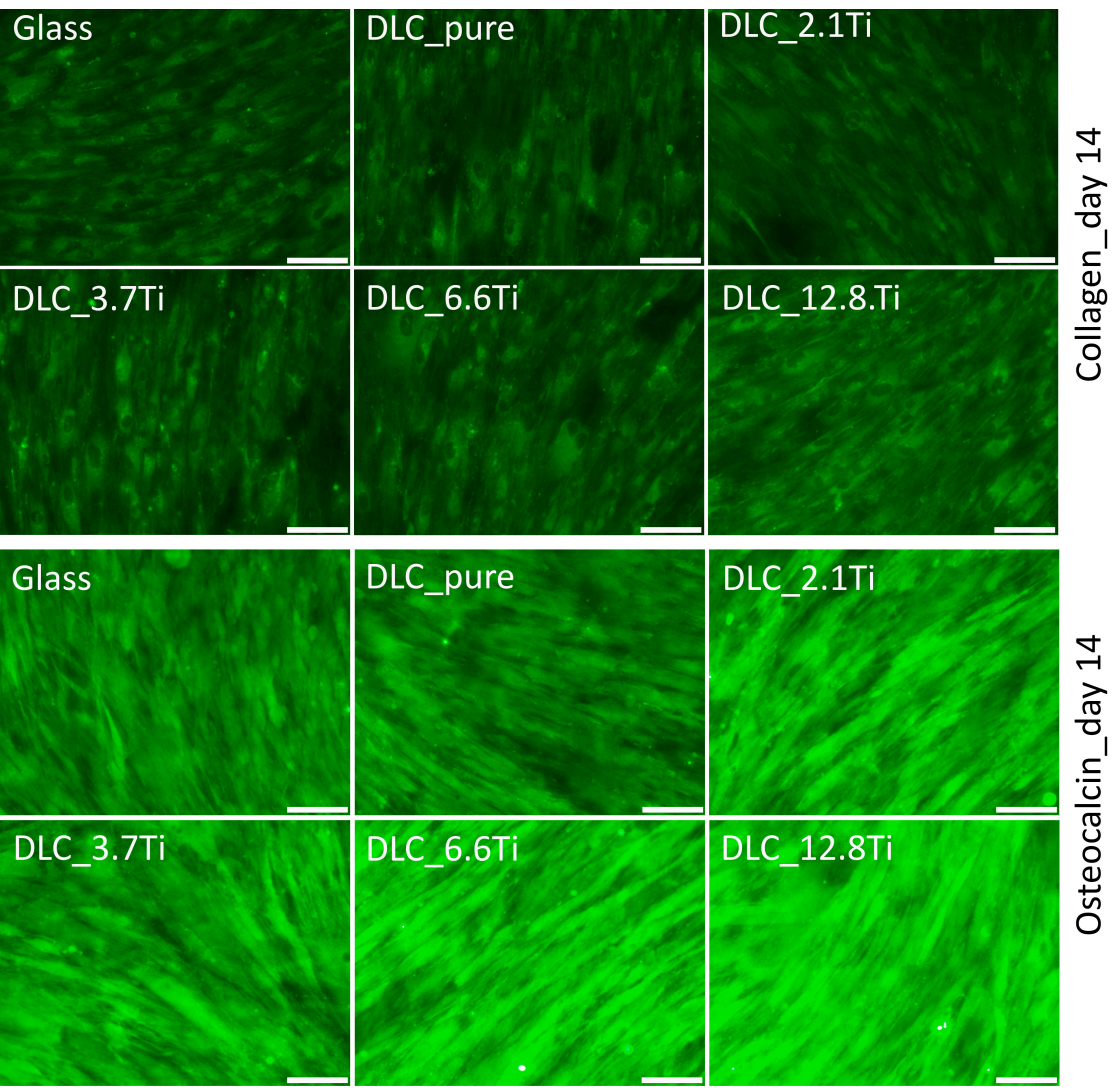
The immunofluorescence staining of type I collagen and osteocalcin in MSCs on glass, on pure DLC, and on DLC doped with Ti (2.1, 3.7, 6.6, and 12.8 at.% of Ti) on day 14. Representative images were selected. Olympus IX71 microscope, IX71 digital camera, objective ×20, scale bar = 100 µm.

Immunofluorescence staining was used to visualize type I collagen (i.e., an early marker of osteogenic differentiation) and osteocalcin (i.e., a late marker of osteogenic differentiation) on days 14 and 21 of the culture. The presence of type I collagen was detectable in ADSCs both on day 14 (the first interval of staining) and day 21 (the last interval of staining) (Figure 9). On day 7, the deposition of type I collagen in ADSCs was initially observable mainly intracellularly (Figure S2); however, on day 14 and especially on day 21, its extracellular deposition and fibril formation were noticeable (Figure 9 and Figures S3 and S4). The ADSCs cultured on all studied samples were positively stained for osteocalcin. The staining of osteocalcin on day 7 showed its preferential localization in the nuclear region of the cells (Figure S2). In comparison with this, on days 14 and 21, cytoplasmic localization was observed (Figure 10 and Figures S5 and S6). Similarly, staining of type I collagen revealed a strong signal in bmMSCs on day 14 and also on day 21 (Figure 11 and Figures S7 and S8). The fibrillar arrangement of type I collagen was not as pronounced as in the case of ADSCs; however, a strong diffuse signal was detected in cells on all tested samples. The bmMSCs were also positively stained for osteocalcin in both time intervals on all tested samples, with the strongest signal on both DLC_12.8Ti and DLC_6.6Ti samples (Figure 11 and Figures S9 and S10).

**Figure 12:**
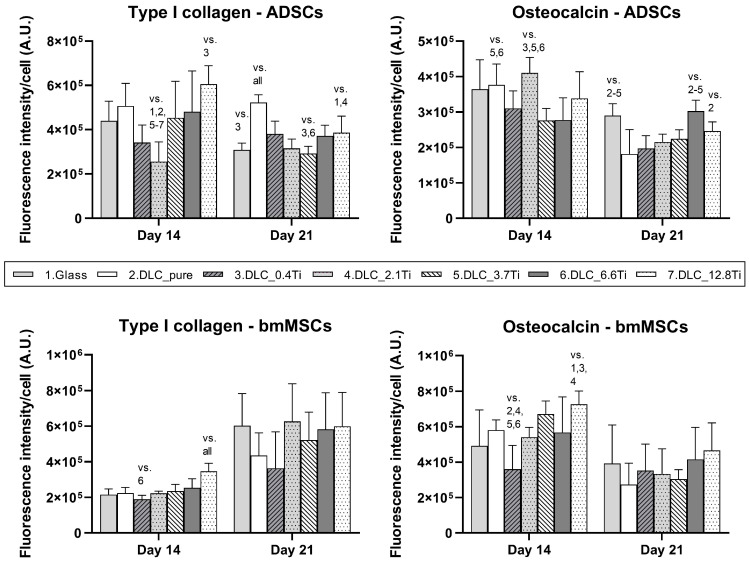
The fluorescence intensity of type I collagen and osteocalcin in ADSCs and bmMSCs on glass, on pure DLC, and on DLC doped with Ti (0.4, 2.1, 3.7, 6.6, and 12.8 at.% of Ti) on days 14 and 21. The fluorescence intensity is related to the cell number. The data are expressed as mean + SD. ANOVA, Holm–Sidak method. The statistical comparison among the samples was made on cells on the same day of the culture. The statistical significance (*p* ≤ 0.001) is specified above the columns by the numbers of the compared experimental groups; vs. “all” means that a given value is significantly different from all values in the same time interval.

The results for the fluorescence intensity of osteogenic markers are summarized in Figure 12. The lowest intensity of type I collagen in ADSCs was detected on DLC_0.4Ti and DLC_2.1Ti on day 14. Similar results were obtained on day 14 for bmMSCs, where the intensity of type I collagen was highest on DLC_12.8Ti. Nevertheless, on day 21, no clear trend in the fluorescence intensity of type I collagen depending on the amount of Ti was observed. The fluorescence intensity of osteocalcin was slightly higher on DLC-based materials with a higher Ti content on day 14 (in bmMSCs) and on day 21 (in ADSCs). Although the absolute values of fluorescence intensity per cell decreased slightly between days 14 and 21 in most of the osteogenic markers, a strong signal of the markers was still detected in cells on all studied samples on day 21 (Figure 12).

#### 2.2.4. Measurements of ALP Activity

**Figure 13:**
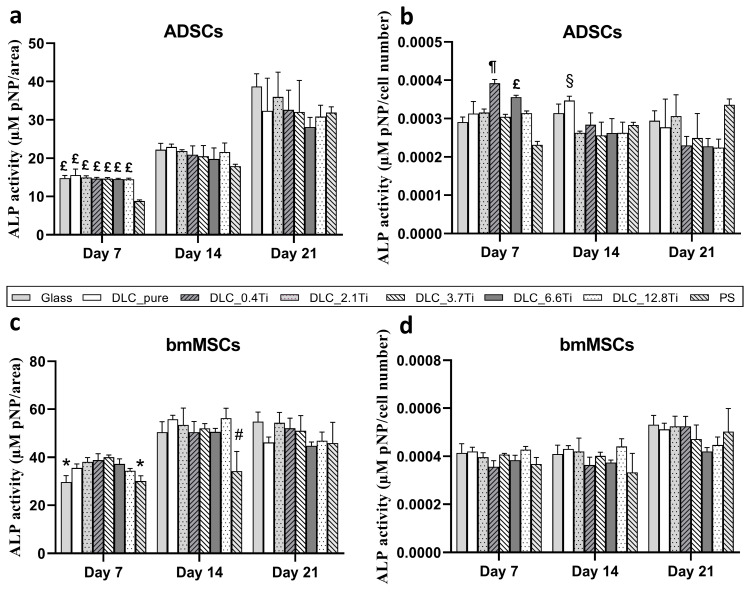
The ALP activity of ADSCs (**a**,**b**) and bmMSCs (**c**,**d**) on glass, on pure DLC, on DLC doped with Ti (0.4, 2.1, 3.7, 6.6, and 12.8 at.% of Ti), and on polystyrene (PS) on days 7, 14, and 21 normalized per area (**a**,**c**) and per cell number (**b**,**d**). The data are expressed as mean + SD. ANOVA, Student–Newman–Keuls method. The statistical comparison among the samples was made on cells on the same day of the culture. The statistical significance (*p* ≤ 0.001) is specified above the columns (* means a lower value than on 0.4, 2.1, and 3.7 at.%; # means a lower value than on pure DLC and 2.1 and 12.8 at.%; ¶ means a higher value than on glass and PS; £ means a higher value than on PS; § means a higher value than on 0.4, 3.7, and 12.8 at.%).

In addition to the immunofluorescence staining of type I collagen and osteocalcin, the osteogenic differentiation of cells, supported by the addition of specific osteogenic supplements from day 4 of the culture, was observed. Potential differences among the tested samples were evaluated using the ALP activity assay on days 7, 14, and 21. The results were normalized to the area of the samples and/or to the cell number. The ALP activity/area increased in time both in ADSCs and in bmMSCs (Figure 13a,c). Only mild differences were observed among the samples. Specifically, control PS showed lower initial ALP activity/area values on days 7 and 14 in comparison with the DLC samples. However, this trend disappeared on day 21. Concerning the recalculation of ALP activity to the cell number, both ADSCs and bmMSCs showed a relatively stable trend in ALP activity within the whole cell culture period. Almost no significant differences were measured among the studied samples (Figure 13a–d).

#### 2.2.5. Measurements of Calcium Deposition

**Figure 14:**
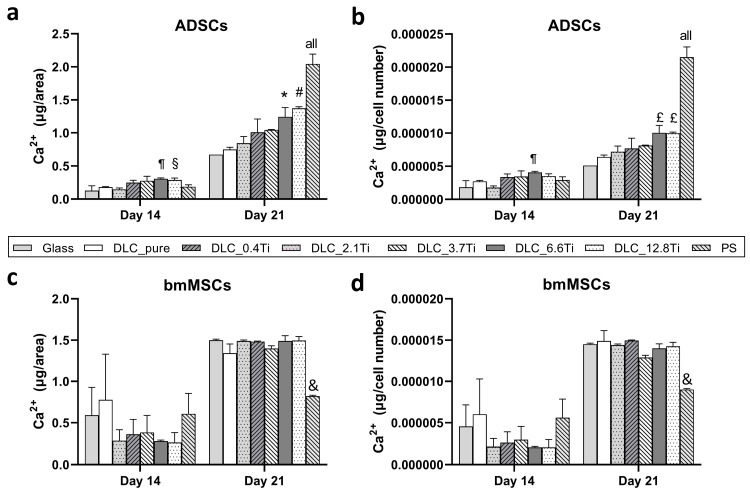
The calcium deposition of ADSCs (**a**,**b**) and bmMSCs (**b**,**c**) on glass, on pure DLC, on DLC doped with Ti (0.4, 2.1, 3.7, 6.6, and 12.8 at.% of Ti), and on polystyrene (PS) on days 14 and 21 normalized per area (**a**,**c**) and per cell number (**b**,**d**). The data are expressed as mean + SD. ANOVA, Student–Newman–Keuls method. The statistical comparison among the samples was made on cells on the same day of the culture. The statistical significance (*p* ≤ 0.05) is specified above the columns (¶ means a higher value than on glass and 0.4 at.%; § means a higher value than on glass; * means a higher value than on glass, pure DLC, and 0.4 at.%; # means a higher value than on glass, pure DLC, and 0.4–3.7 at.%; £ means a higher value than on glass and pure DLC; & means a statistically different value from glass, DLC_pure, DLC_0.4Ti, DLC_2.1Ti, DLC_3.7Ti, DLC_6.6Ti, and DLC_12.8Ti; “all” means that a given value is significantly different from all values in the same time interval).

To observe late osteogenic differentiation, a calcium deposition assay was performed on day 14 and day 21 after seeding. On all studied samples, the calcium deposition increased between days 14 and 21 in both cell models (i.e., ADSCs and bmMSCs) (Figure 14a–d). Interestingly, a different trend in calcium deposition in ADSCs and bmMSCs was observed among the studied samples. Specifically, on day 14 in ADSCs, there was greater calcium deposition (*p* < 0.05) on DLC containing a greater amount of Ti (i.e., DLC_6.6Ti and DLC_12.8Ti) than on glass, DLC_pure, and DLC_0.4Ti (Figure 14a,b). This trend in ADSCs was even more pronounced on day 21 when increasing calcium deposition values were measured in accordance with the increasing Ti content in all DLC samples. Similar to day 14, these trends reached statistical significance (*p* < 0.05) for DLC_6.6Ti and DLC_12.8Ti vs. glass and DLC_pure. At the same time, however, the highest calcium deposition values were measured in the cells on PS. By contrast, the calcium deposition in bmMSCs scarcely differed among the DLC samples either on day 14 or on day 21, and the lowest values were observed on PS (Figure 14c,d).

## 3. Discussion

### 3.1. Material Preparation and Characterization

Ti-DLC layers are known to possess very good corrosion resistance, high electrochemical stability, and adhesion to the substrate in a simulated body fluid [27]. Additionally, a reduction in friction and wear was reported in comparison with the substrate itself. The layers with a higher carbon content also exhibited superior tribocorrosion properties in a simulated body fluid [27]. In various publications, Ti-DLC layers containing increasing Ti concentrations have been prepared by various techniques, such as dual PLD, PLD combined with magnetron sputtering (MS), dual MS, and plasma-enhanced chemical vapor deposition [15,16,21,28]. The specific preparation technique can influence the results and can make a potential comparison of results among the publications difficult. Specifically, some discrepancies can be observed in the trends in the contact angle, SFE, or hardness values.

In our current study, we prepared and characterized pure DLC layers and Ti-doped DLC layers containing from 0.4 to 12.8 at.% of Ti by dual PLD. The preparation and characterization of similar Ti-DLC layers for future application as implant coatings were previously described and discussed in our earlier studies [16,21]. Concerning analyses of material characterization prior to cell seeding, our current results show similar trends as in our previous publications [16,21]. Specifically, increasing contact angle, slightly increasing roughness, and more numerous droplets (mainly around 10 μm in size) were present on the surface with increasing Ti content. Similar results were observed by Zhang et al. [15] and by Zhao et al. [28], where the increasing Ti content in DLC layers, prepared by the dual MS and plasma-enhanced chemical vapor deposition technique, caused increasing Ra and Rq values. In contrast, Zhao et al. [28] described a slightly decreasing contact angle with increasing Ti content (1.3–3.2 at.% of Ti) in Ti-DLC layers. Such discrepancies could have been caused by different fabrication techniques for layers with different properties. Interestingly, from our previous results [21] and also from our current results, it seems that the samples with concentrations from 2.1 at.% to 12.8 at.% of Ti showed quite similar properties regarding the contact angle and SFE measurements. The measured values between 2.1 and 12.8 at.% of Ti differed only slightly in comparison to DLC_pure and DLC_0.4Ti. In contrast, sample DLC_0.4Ti showed significantly different values from DLC_pure and DLC doped with a higher Ti content, in both SFE and contact angle values. The differences in the absolute values of surface topography, contact angle, SFE, and chemical composition that occur between samples previously characterized and currently characterized may have been caused by a different batch of materials and by different testing devices (e.g., for XPS, SEM, and AFM) [16,21]. However, although the absolute values measured in various analyses differ, the trends are the same in all our publications.

Concerning the mechanical properties of Ti-DLC layers prepared by dual PLD, increasing Ti concentration significantly decreased the hardness from 31.2 GPa (for pure DLC) to 10.4 GPa (for DLC containing 12.8 at.% of Ti) and decreased the Young’s modulus in a similar manner, i.e., from 263 GPa (for pure DLC) to 134 GPa (for DLC containing 12.8 at.% of Ti) [16]. At the same time, it increased the critical adhesion force Lc3 from 9.9 N (for pure DLC) to 18.4 N (for DLC containing 12.8 at.% of Ti) [16]. Similar trends in hardness and critical adhesion force values can be observed for Ti-DLC layers prepared by PLD combined with MS [16]. From this point of view, Ti doping of DLC layers approximated hardness and Young’s modulus values closer to healthy cortical bone, which is very desirable for potential applications in implant orthopedic surgery.

### 3.2. Biocompatibility—Cell Adhesion, Proliferation, and Differentiation

The set of experiments was performed with two cell types that are important for bone regeneration in vivo and for bone tissue engineering in general. The osteogenic differentiation of ADSCs and bmMSCs, which was supported by the appropriate composition of the medium, was successfully obtained during the experiments on all observed samples (i.e., pure DLC and Ti-doped DLC layers, control glass, and PS samples). The osteogenic differentiation of bmMSCs and ADSCs by a similar medium composition was proved in our previous publications [29,30]. In our current results, slight differences between bmMSCs and ADSCs were observed in terms of proliferation potential, the onset of osteogenic differentiation, and dependence on Ti content. Specifically, the bmMSCs rapidly proliferated in the initial phase of the experiments (i.e., up to day 14) and reached full confluence followed by the formation of another cell layer shortly afterward. Moreover, they reached the highest ALP activity values on day 14, and these values were similar or slightly decreased on day 21. In contrast, the ADSCs showed continuous proliferation, which was accompanied by full confluence up to day 21. In addition, the ADSCs showed increasing ALP activity up to day 21. In both cell types, an increasing amount of Ca was detected up to day 21, and the maximum fluorescence intensity per cell in most of the osteogenic markers was detected on day 14. These findings can indicate an earlier onset of the proliferation and differentiation phase in bmMSCs than in ADSCs. The results are also in accordance with our previous research [29], where bmMSCs were better spread, strongly attached, and showed higher proliferation and differentiation capacity than ADSCs when chemically differentiated toward osteoblasts on composite chitosan/β-1,3-glucan/hydroxyapatite scaffolds. Interestingly, the bmMSCs and ADSCs in some current analyses acted slightly differently depending on the amount of Ti in the DLC layers. The metabolic activity of ADSCs showed increasing values with increasing amounts of Ti on days 7, 14, and 21, and the same trend was obtained for the amount of Ca, especially on day 21. In contrast, the proliferation and osteogenic differentiation of bmMSCs did not show crucial dependency on the amount of Ti, except for the fluorescence intensity of type I collagen on day 14. These results are in accordance with the behavior of bmMSCs on variably Cr-doped DLC layers prepared by simultaneous PLD and MS, where neither the metabolic activity of the cells nor the ALP activity or Ca deposition in the cells were changed depending on the amount of chromium [30]. In addition, Zhang et al. tested the biocompatibility of human periodontal ligament cells on similar Ti-doped DLC layers (Ti content ranging from 0 to 26.98 at.%), and they described favorable initial biocompatibility on all samples regardless of the Ti content [15]. Similarly, our previous study on the interaction between Saos-2 cells and Ti-doped DLC layers (containing 0.4–12.8 at.% Ti) showed a very similar proliferation rate on all Ti-doped DLC layers. Only the formation of focal adhesion and the cell spreading area was influenced by the amount of Ti, i.e., a greater amount of Ti caused more pronounced focal adhesions and a greater cell spreading area [16,21]. In a similar way, our current study proved that there were more pronounced focal adhesions and a better-developed F-actin cytoskeleton in ADSCs cultured on DLC-based layers containing a higher Ti content. The development of the F-actin cytoskeleton and the formation of focal adhesions during the initial cell adhesion to the material surface is also related to the surface topography, the wettability, and the electrical charge and conductivity [31,32]. From this point of view, the better formation of focal adhesions in Saos-2 and ADSCs on Ti-DLC layers with a higher Ti content could be influenced mainly by slightly higher surface roughness and a higher concentration of both Ti and O than in pure DLC layers and in Ti-DLC layers containing a lower at.% of Ti. In addition, it is known that other types of stem cells or various mature cells can act differently in terms of cell adhesion, proliferation, and differentiation on similar material surfaces [33,34]. Our results could indicate different sensitivity of different cell types to the material surface chemistry/surface topography, which is influenced by the Ti content. Nevertheless, the biocompatibility and the differentiation parameters in the cells were still at high levels on all tested samples in our experiments.

The fast proliferation and an early onset of osteogenic differentiation of mesenchymal stem cells is crucial for patients’ bone healing; therefore, data from shorter time intervals, i.e., days 7 and 14, are the most important. It is also worth mentioning that during the biological experiments lasting three weeks, it was possible to observe sporadic detachment of DLC_pure layers from glass substrates on some samples. This detachment proved lower adhesion of pure DLC layers to the substrate, as was previously mentioned in the Introduction. This could affect, e.g., the measurements of the metabolic activity of the ADSCs on day 21 for these samples.

An increasing Ti content in DLC layers not only influences the mechanical properties, surface topography, and chemical composition of the layers, which is subsequently important for cell–material interactions but can also reduce bacterial adhesion to Ti-DLC coatings [28]. Specifically, an increase in concentration from 1.3 to 3.2 at.% of Ti significantly reduced the adhesion of *Escherichia coli* and *Pseudomonas aeruginosa* in comparison with uncoated stainless steel and in comparison with a pure DLC layer [28]. Such properties are very favorable for orthopedic metal implants, as they contribute to better osseointegration and lower potential implant loss, which is often caused by infectious pathogens.

## 4. Materials and Methods

### 4.1. Material Preparation

**Figure 15:**
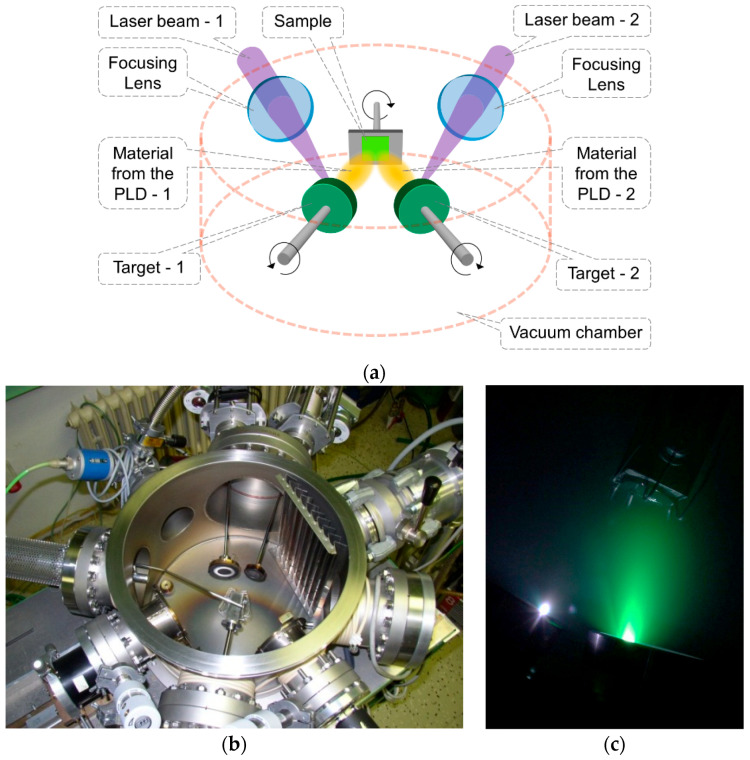
(**a**) Scheme of dual PLD using two lasers and two targets, (**b**) photo of the deposition chamber, and (**c**) photo of the deposition process.

Pure DLC layers and Ti-doped DLC layers were prepared by dual pulsed laser deposition (PLD) (see Figure 15a–c), which has been previously described [16,21]. In brief, DLC layers were deposited on glass substrates (dimensions: 1 cm × 1 cm × 0.1 cm) by a dual PLD method using two excimer KrF lasers (details of the laser rate are listed in Table 2). The DLC layers were doped with variable amounts of Ti. The increasing Ti content was as follows: 0.4, 2.1, 3.7, 6.6, and 12.8 at.% of Ti (These values were measured by XPS immediately after dual PLD and are used as a default to name the samples in this study). The Ti-DLC layers were 120 nm in thickness.

### 4.2. Material Characterization

#### 4.2.1. Scanning Electron Microscopy (SEM)

The material surface morphology (micro-scale) was determined by a scanning electron microscope (SEM) using LYRA3 (Tescan, Brno, Czech Republic). The applied acceleration voltage for SEM was 10 kV. The substrates were covered with a Pt conductive layer with a thickness of 10 nm using a diode sputtering method (Quorum Q300T, Quorum Technologies, Laughton, UK).

#### 4.2.2. Atomic Force Microscopy (AFM)

The material surface morphology (nanoscale) was examined with a Dimension Icon atomic force microscope (Bruker Corp., Brno, Czech Republic). The ScanAsyst mode in Air was used. A SCANASYST-AIR Silicon Tip on a Nitride Lever with a spring constant of 0.4 N/m was used. NanoScope Analysis software (http://nanoscaleworld.bruker-axs.com/nanoscaleworld/media/p/141.aspx, accessed on 24 January 2024) was applied for data processing. The mean roughness values (Ra) represent the average of the deviations from the center plane of the sample.

#### 4.2.3. Wettability

The material surface wettability was determined by goniometry using the static water drop method. The measurements of water contact angles were performed using the DSA100 Drop Shape Analyzer (A.KRÜSS Optronic, Hamburg, Germany). Drops of distilled water or glycerol (volume 5.0 μL) were deposited at 5 different positions on the sample, and the consequent photo was evaluated. DSA4 software v1.41r was used to evaluate the drops. The Owens–Wendt–Rabel–Kaelble calculation method was used to determine the surface free energy values (SFE).

#### 4.2.4. X-ray Photoelectron Spectroscopy (XPS)

The elemental concentration was determined by X-ray photoelectron spectroscopy (XPS). An Omicron Nanotechnology ESCAProbeP spectrometer (Omicron Nanotechnology Ltd, Taunusstein, Germany) was used. The exposed and analyzed area had dimensions of 2 × 3 mm^2^. The X-ray source was monochromatic at 1486.7 eV. The take-off angles were 0° and 81° to the perpendicular of the surface of the sample. The atomic concentrations of the elements were determined by the CASA XPS program using an integrated area of spectrum lines and relative sensitivity factors that are quoted in the database of CASA XPS. The error of determination was ±1.5%.

#### 4.2.5. Energy-Dispersive X-ray Spectroscopy (EDS)

For elemental concentration, EDS was used on the Quanta 450 scanning electron microscope (FEI, Hillsboro, OR, USA) equipped with a Si(Li) Apollo XL Silicon Drift Detector with an FET preamplifier (EDAX Inc., Mahwah NJ, USA) at a magnification of 1000× and an accelerating voltage of 12.5 kV. Prior to the EDS analyses, samples were attached by carbon tape to an alumina stub and were gold-coated on a K550X (Quorum Technologies Ltd, Ashford, UK) sputter coater in an argon atmosphere. The weight and the atomic fractions of the chemical elements were characterized. Five measured fields were taken for each sample at a magnification of 1000× (measured area of 85.456 µm^2^, i.e., a total of 427.331 µm^2^ for each sample). On each sample, the EDS map was taken at 1000× magnification. Data acquisition was performed on EDAX TSL OIM software, v. 7.0 with ZAF corrections.

### 4.3. Biological In Vitro Experiments

#### 4.3.1. Cell Models

Human adipose tissue-derived stem cells (ADSCs) were isolated from a lipoaspirate using collagenase digestion; the detailed isolation procedure was described in our earlier studies [29]. The isolation was conducted according to the tenets of the Declaration of Helsinki and was approved by the Ethics Committee at Na Bulovce Hospital in Prague (approval issued on 21 August 2014; No. 11.6.2019/9150/EK-Z, 2019, and No. 16.3.2023/10795/EK-Z, 2023). Written informed consent was obtained from the donor (a healthy 46-year-old woman) before the liposuction procedure was applied (thigh region of liposuction, negative pressure of −200 mmHg during liposuction). After the isolation procedure, the cells were expanded in Dulbecco’s modified Eagle’s Medium (DMEM) (Gibco, Thermo Fisher Scientific) supplemented with 10% (vol/vol) of fetal bovine serum (FBS) (Gibco, Thermo Fisher Scientific), 10 ng/mL of basic fibroblast growth factor (FGF2) (GenScript, Rijswijk, Netherlands), and 40 μg/mL of gentamicin (LEK, Ljubljana, Slovenia). The cells were further characterized by flow cytometry (Accuri C6 Flow Cytometer, BD Biosciences, San José, CA, USA) for the presence of specific CD markers. The analysis revealed a high presence of CD105 (99.9%), CD90 (99.5%), CD73 (100%), and CD29 (100%) and a very low presence of CD146 (4.7%), CD45 (3.8%), CD34 (0.2%), and CD31 (0.5%).

Bone marrow mesenchymal stem cells (bmMSCs) originating from a single donor were purchased from ScienCell Research Laboratories (Cat. No. 7500, ScienCell Research Laboratories, Carlsbad, CA, USA). Before the experiments, the cells were expanded in Mesenchymal Stem Cell Medium (MSCM) (Cat. No. 7501, ScienCell Research Laboratories) in 75 cm^2^ tissue culture flasks (TPP, Trasadingen, Switzerland) coated with poly-L-lysine (15% vol/vol in H20; Cat. No. P4707, Sigma-Aldrich, St. Louis, MO, USA).

#### 4.3.2. Culture Conditions

**Scheme 1:**
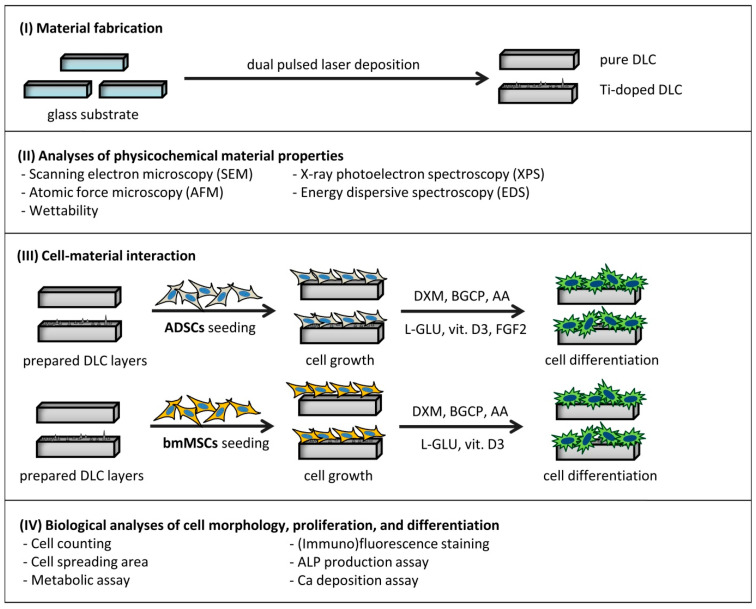
Scheme of the experimental work.

An overview of the biological experiments is illustrated in Scheme 1. Prior to cell seeding, the tested samples (i.e., glass, DLC_pure, DLC_0.4Ti, DLC_2.1Ti, DLC_3.7Ti, DLC_6.6Ti, and DLC_12.8Ti) were disinfected with 70% ethanol for 1 h at room temperature (RT). The samples were further washed with PBS and placed in 24-well tissue culture test plates (TPP, Trasadingen, Switzerland). ADSCs (passage 3) were seeded on the samples at a density of 14,000 viable cells/cm^2^ in 1.5 mL of DMEM supplemented with 10% (vol/vol) of FBS, 10 ng/mL of FGF2, and 40 μg/mL of gentamicin. From day 4, this growth medium was replaced with an osteogenic medium, i.e., DMEM supplemented with 10% (vol/vol) FBS, 10 nM of dexamethasone (DXM) (Cat. No. D2915, Sigma-Aldrich), 10 mM of β-glycerophosphate (BGCP) (Cat. No. G9422, Sigma-Aldrich), 50 μg/mL of ascorbic acid (AA) (Cat. No. 49752-10G, Sigma-Aldrich), 2 mM of L-Glutamine (L-Glu) (G-8540, Sigma-Aldrich), 1 μM of dihydroxyvitamin D3 (vit.D3) (D1530, Sigma-Aldrich), 5 ng/mL of FGF2, and 40 μg/mL of gentamicin. The ADSCs were cultured at 37 °C in a humidified air atmosphere with 5% CO_2_ for 21 days in total, and the osteogenic medium was replaced twice per week.

The bmMSCs (passage 3) were seeded on the samples at a density of 5000 viable cells/cm^2^ in 1.5 mL of α-MEM (Cat. No. 11900-016, Gibco, Thermo Fisher Scientific) supplemented with 15% (vol/vol) of FBS and 40 μg/mL of gentamicin. From day 4, this growth medium was exchanged with an osteogenic medium, i.e., α-MEM supplemented with 15% (vol/vol) of FBS, 10 nM of DXM, 10 mM of BGCP, 50 μg/mL of AA, 2 mM of L-GLU, 1 μM of vit. D3, and 40 μg/mL of gentamicin. The bmMSCs were cultured at 37 °C in a humidified air atmosphere with 5% CO_2_ for 21 days in total, and the osteogenic medium was replaced twice per week.

#### 4.3.3. Image Analysis—Cell Numbers and Cell Morphology

On day 1, the cell spreading area of 67 to 106 (ADSCs) and of 59 to 103 (bmMSCs) separate cells, i.e., cells without cell–cell contacts, which had been fixed and stained with Texas Red C_2_-maleimide (concentration in PBS of 10 ng/mL, incubation for 1 h), was counted on microphotographs using TESCAN Atlas software, version 2.9.5.2. (Tescan s.r.o. Digital microscopy Imaging, Brno, Czech Republic)

On days 1, 7, 14, and 21, the cell numbers were manually counted on microphotographs according to the Hoechst 33258 staining of cell nuclei. Between 10 and 20 microphotographs for each sample in each time interval were analyzed. From these data, growth curves were constructed.

#### 4.3.4. Metabolic Activity of the Cells

On days 1, 7, 14, and 21 of the culture, in addition to the direct cell counting, the cell proliferation was estimated based on the metabolic activity of the cells. The metabolic activity was evaluated by resazurin (Cat. No. R7017, Sigma-Aldrich, St. Louis, MO, USA) conversion, which is brought about by the mitochondrial enzymes of the cells. A detailed description of the method was presented in our previous article [30]. In brief, the resazurin solution (40 μmol/L) in DMEM without phenol red was prepared and added to the cells that had previously been washed with PBS. The cells with the solution were incubated at 37 °C for 3 h and 30 min (ADSCs) or for 2 h (bmMSCs) on all days of the evaluation. The fluorescence of the solution was subsequently measured (Ex/Em = 530/590 nm) and was corrected to the background control (resazurin solution without cells).

#### 4.3.5. (Immuno)fluorescence Staining

(Immuno)fluorescence staining was used to visualize the markers of cell adhesion, growth, and differentiation on the tested materials. Prior to all types of staining, the cells were washed with PBS and were fixed with 4% paraformaldehyde (10 min). Subsequently, the cells were pre-treated with PBS containing 1% (*w*/*v*) bovine serum albumin (BSA) (Sigma-Aldrich) and 0.1% (vol/vol) TritonX-100 for 20 min and were further pre-treated for 20 min with PBS containing 1% (vol/vol) of Tween 20. After each step, the samples with cells were washed with pure PBS. The list of fluorescent dyes and the primary and secondary antibodies used in the experiments are shown in Table 3. On days 14 and 21, the fluorescence intensity of osteogenic markers (i.e., type I collagen and osteocalcin) was quantified from fluorescent micrographs (8–12 per sample) using ImageJ. The images were taken using the same exposure time of the specific channel, i.e., green or red, for all samples and also for negative controls stained similarly without the usage of primary antibody. The intensity measurement was performed via ImageJ 1.54f software (Wayne Rasband and contributors, National Institutes of Health, Stapleton, NY, USA) by measuring integrated intensity. The final intensity for each image was calculated by subtraction of the negative control and then the value was normalized to the cell nuclei number for each image.

#### 4.3.6. ALP Activity Assay

For a study of the early and/or the mid-term phase of the osteogenic differentiation of cells, the activity of ALP was measured on days 7, 14, and 21. The method was described in detail in our previous article [30]. In brief, 0.1 mg/mL p-nitrophenyl phosphate in the substrate buffer (50 mmol/L glycine, 1 mmol/L MgCl_2_, pH 10.5; Sigma-Aldrich) was added to the samples with cells. The cells were incubated with the solution for 10 min at RT; then, the solution was added to an equal amount of NaOH (1 mol/L), and the absorbance of the samples was measured (λ = 405 nm). The results were normalized to the area of the samples and to the cell number on the same day of the culture.

#### 4.3.7. Calcium Colorimetric Assay

To detect a late phase of osteogenic differentiation of cells and mainly the deposition of inorganic compounds by the cells, a calcium colorimetric assay was performed on days 14 and 21. The method was described in detail in our previous article [30]. In brief, the samples with cells were dried and treated with HCl (0.5 mol/L) overnight. Then, the cell suspensions were centrifuged, and the supernatants that were obtained were further analyzed using a calcium colorimetric assay (Cat. No. MAK022-1KT, Sigma-Aldrich) according to the manufacturer’s protocol. Similar to the ALP activity assay, the results were normalized to the area of the samples and to the cell number on the same day of the culture.

#### 4.3.8. Statistical Analysis

The data are presented as the arithmetical mean unless otherwise specified, and the number of samples for each analysis used in this work is specified in the relevant chapter of the Section 4 or in the relevant figure caption.

For an evaluation of the contact angle and the total surface free energy, a One-Way Analysis of Variance and multiple comparisons versus the control group (Bonferroni *t*-test) were used (SigmaPlot 14.0). The statistical analysis of the EDS evaluation was performed in GraphPad Prism (ver. 9.5.0 (730), GraphPad Software, San Diego, CA, USA). For the evaluation of the EDS data, non-parametric analysis was used since the assumption of normality (Shapiro–Wilk test and the construction of Q-Q plots) or homoscedasticity (Levene’s test) was violated. A two-tailed Mann–Whitney test was applied to compare each material separately with the control sample (DLC_pure). Statistical significance was accepted at *p* ≤ 0.05. Scatter plots with the median and the interquartile range (IQR) plotted were used for the graphical presentation of the data.

For all the biological experiments, the data are expressed as mean + SD, and for the statistical analyses, the One-Way ANOVA, Student–Newman–Keuls method was applied, unless otherwise specified in the figure caption. The statistical comparison was made on cells on the same day of the culture, and *p* ≤ 0.05 was considered significant. Due to the large number of compared samples, some plots contain significance *p* ≤ 0.001 only. Statistical analyses were performed using SigmaStat 3.5 software (Systat Software Inc., Palo Alto, CA, USA) and the data were plotted using GraphPad Software v. 10.2.0 (Boston, MA, USA).

## 5. Conclusions

In the present study, Ti-doped DLC layers with variable Ti content were prepared by dual pulsed laser deposition, and the cytocompatibility of the layers was proved in vitro using two mesenchymal stem cell types: ADSCs and bmMSCs. A complex comparative study of the two mesenchymal stem cell types was focused on material properties, cell adhesion, proliferation, and osteogenic differentiation.

The physicochemical characterization of Ti-doped DLC layers showed an increased content of both Ti and O, the contact angle, and roughness and decreased SFE with increased Ti concentration from DLC_2.1Ti to DLC_12.8Ti.

Ti-doped DLC layers with their nanostructured surface provided a suitable substrate for initial cell adhesion and spreading, and both ADSCs and bmMSCs maintained their typical spindle-shaped or polygonal-shaped morphology after adhesion and spreading.

The cell proliferation, measured as the density of the cell population and the metabolic activity of the cells, of both ADSCs and bmMSCs on Ti-DLC layers, was similar or even higher than on control glass and PS during the three-week cell culture.

The ADSCs and bmMSCs were successfully differentiated toward osteoblasts on all tested materials. The Ti levels did not significantly influence the rate of osteogenic differentiation in most of the tested parameters, and the differentiation was mainly driven chemically by the composition of the medium.

The comparison between the two cell types revealed that the bmMSCs exhibited greater initial proliferation potential and an earlier onset of osteogenic differentiation, which was proved by greater ALP activity on days 7 and 14, greater osteocalcin production on day 14, and earlier and greater calcium deposition in comparison with ADSCs.

The ADSCs proliferated steadily over a period of 21 days and produced more collagen than the bmMSCs on day 14. In addition, ADSCs seemed to be more sensitive to the Ti content, as ADSCs showed a slightly greater formation of focal adhesions, and evinced greater metabolic activity, collagen production, and Ca deposition with increasing Ti content in the layer. The bmMSCs were less sensitive to Ti content, and more uniform results were obtained.

Based on our results combining the material characterization and in vitro biological analyses, we would suggest using Ti-DLC layers containing 2.1–12.8 at.% of Ti for further in vivo animal studies.

## Figures and Tables

**Table 1:** Contact angle and surface energy values.

Sample	Contact Angle (°)		Surface Energy (mN/m)
	Water	Glycerol	Total
Glass	45.6 ± 2.5 *	37.7 ± 1.2 *	53.0 ± 7.22 *
DLC_pure	76.8 ± 1.4	48.3 ± 5.0	59.0 ± 13.53
DLC_0.4Ti	27.7 ± 3.1 *	55.1 ± 1.0 *	90.3 ± 5.98 *
DLC_2.1Ti	78.0 ± 0.9	77.3 ± 1.9 *	26.6 ± 4.68 *
DLC_3.7Ti	78.4 ± 1.7	77.9 ± 3.0 *	26.4 ± 7.73 *
DLC_6.6Ti	80.5 ± 3.4	68.7 ± 1.2 *	30.1 ± 9.54 *
DLC_12.8Ti	86.3 ± 1.5 *	89.0 ± 3.8 *	21.7 ± 7.68 *

* vs. DLC_pure, *p*-value < 0.05.

**Table 2:** Deposition parameters and the original measured titanium content of the DLC layers [16,21].

Sample	Laser Rate [Hz]		Titanium Content [at.%] ^a^	
	L1	L2	Rc	Sp
DLC_pure	26	-	0.0	0.0
DLC_0.4Ti	26	1	0.4	0.7
DLC_2.1Ti	25	3	2.1	3.3
DLC_3.7Ti	25	5	3.7	5.2
DLC_6.6Ti	26	11	6.6	10.1
DLC_12.8Ti	18	23	12.8	24.5

Laser frequencies (L1 and L2) were used for preparing the layers by a dual PLD method; L1—Laser 1 Complex Freq. (repetition rate of Laser 1—Complex); L2—Laser 2 Lumonics Freq. (repetition rate of Laser 2—Lumonics); Rc—as grown (the concentration of as-received surfaces); Sp—sputtered (the concentration of surfaces sputtered-cleaned by an argon cluster ion beam, thickness ∼ 100 nm, deposition time ∼ 8 min); The Rc values measured by XPS after fabrication were used as a default to name the samples in this study; ^a^—measured by XPS.

**Table 3:** Fluorescent dyes and antibodies used in this study.

Type, Name	Supplier	Cat. No.	Concentration
Texas Red C_2_-maleimide	Invitrogen	T6008	10 ng/mL
Phalloidin-TRITC	Sigma-Aldrich	P1951	5 μg/mL
Hoechst 33258	Sigma-Aldrich	B1155	10 μg/mL
Anti-vinculin (mouse)	Sigma-Aldrich	V9131	1:200
Anti-type I collagen (rabbit)	CosmoBio	LSL-LB-1197	1:200
Anti-type I collagen (mouse)	Sigma-Aldrich	C2456	1:200
Anti-osteocalcin (rabbit)	Peninsula Laboratories	T-4743	1:200
Anti-rabbit IgG-Alexa Fluor 488	Thermo Fisher Scientific	A11070	1:400
Anti-rabbit IgG-Alexa Fluor 546	Thermo Fisher Scientific	A11010	1:400
Anti-mouse IgG-Alexa Fluor 488	Thermo Fisher Scientific	A11017	1:400
Anti-mouse IgG-Alexa Fluor 546	Thermo Fisher Scientific	A11003	1:400

## Data Availability

The data presented in this study are available at 10.5281/zenodo.10708675.

## Supplementary Materials

The following supporting information can be downloaded at: https://www.mdpi.com/article/10.3390/ijms25052837/s1.
