# Supplementary material for: Titanium-Doped Diamond-like Carbon Layers as a Promising Coating for Joint Replacements Supporting Osteogenic Differentiation of Mesenchymal Stem Cells
Source: Int J Mol Sci. 2024 Feb 29;25(5):2837. doi: 10.3390/ijms25052837 (PMC10932162; doi:10.3390/ijms25052837)
Supplement: Supplementary file 1 [file ijms-25-02837-s001.zip › ijms-2866232-supplementary.pdf]

Supplementary data

Titanium-doped diamond-like carbon layers as a promising coating for joint replacements supporting osteogenic differentiation of mesenchymal stem cells

Martina Travnickova [1], Elena Filova [1,2*], Petr Slepicka [3] , Nikola Kasalkova Slepickova [3] , Tomas Kocourek [,4,5], Margit Zaloudkova [6], Tomas Suchy [6] and Lucie Bacakova [1,2]

1. aboratory of Biomaterials and Tissue Engineering, Institute of Physiology of the Czech Academy of Sciences, Videnska 1083, 142 00, Prague 4, Czech Republic; martina.travnickova@fgu.cas.cz, elena.filova@fgu.cas.cz, lucie.bacakova@fgu.cas.cz
2. Faculty of Materials and Technology, VSB-Technical University of Ostrava, 17. listopadu 2172/15, 708 00 Ostrava-Poruba, Czech Republic
3 Department of Solid State Engineering, University of Chemistry and Technology, Technicka 5, 166 28, Prague 6, Czech Republic; Petr.Slepicka@vscht.cz, nikola.kasalkova@vscht.cz
4 Institute of Physics of the Czech Academy of Sciences, Na Slovance 2, 182 21 Prague 8, Czech Republic; kocourek@fzu.cz
5 Faculty of Biomedical Engineering, Czech Technical University in Prague, Nam. Sitna 3105, 27 201 Kladno, Czech Republic
6 Institute of Rock Structure and Mechanics, Czech Academy of Sciences, V Holesovickach 94/41, 182 09, Prague 8, Czech Republic; suchyt@irsm.cas.cz, zaloudkova@irsm.cas.cz
* Correspondence: elena.filova@fgu.cas.cz; Tel.: 00420 2 9644 3742

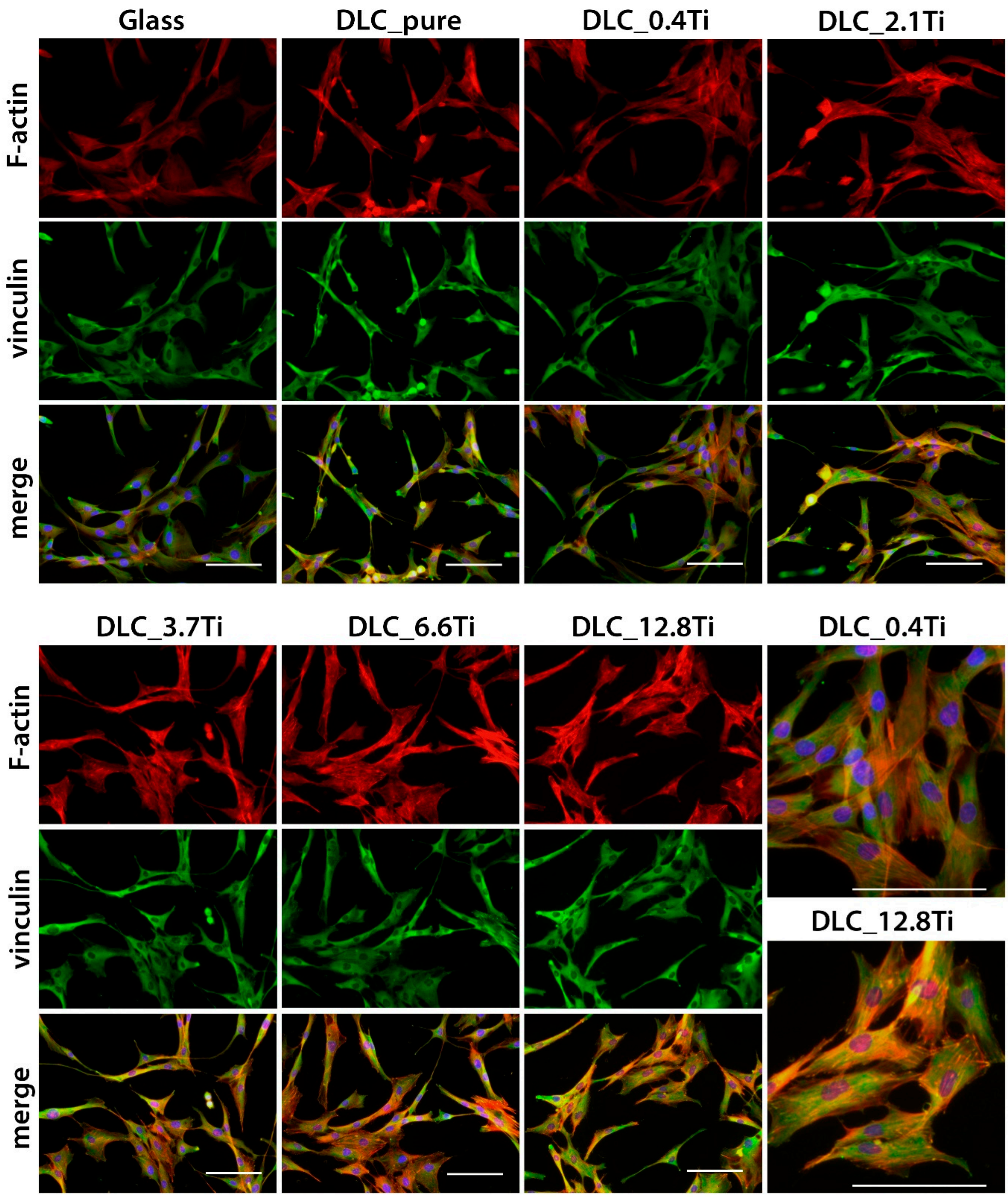

Figure S1. The fluorescence staining of F-actin (red) and vinculin (green) and merged images in ADSCs on glass, on pure DLC, on DLC doped with Ti (0.4, 2.1, 3.7, 6.6, 12.8 at. % of Ti) on day 3. Cell nuclei are counterstained with Hoechst 33258 (blue). Representative images were selected. Olympus IX71 microscope, IX71 digital camera, objective ×20, scale bar = 100 μm.

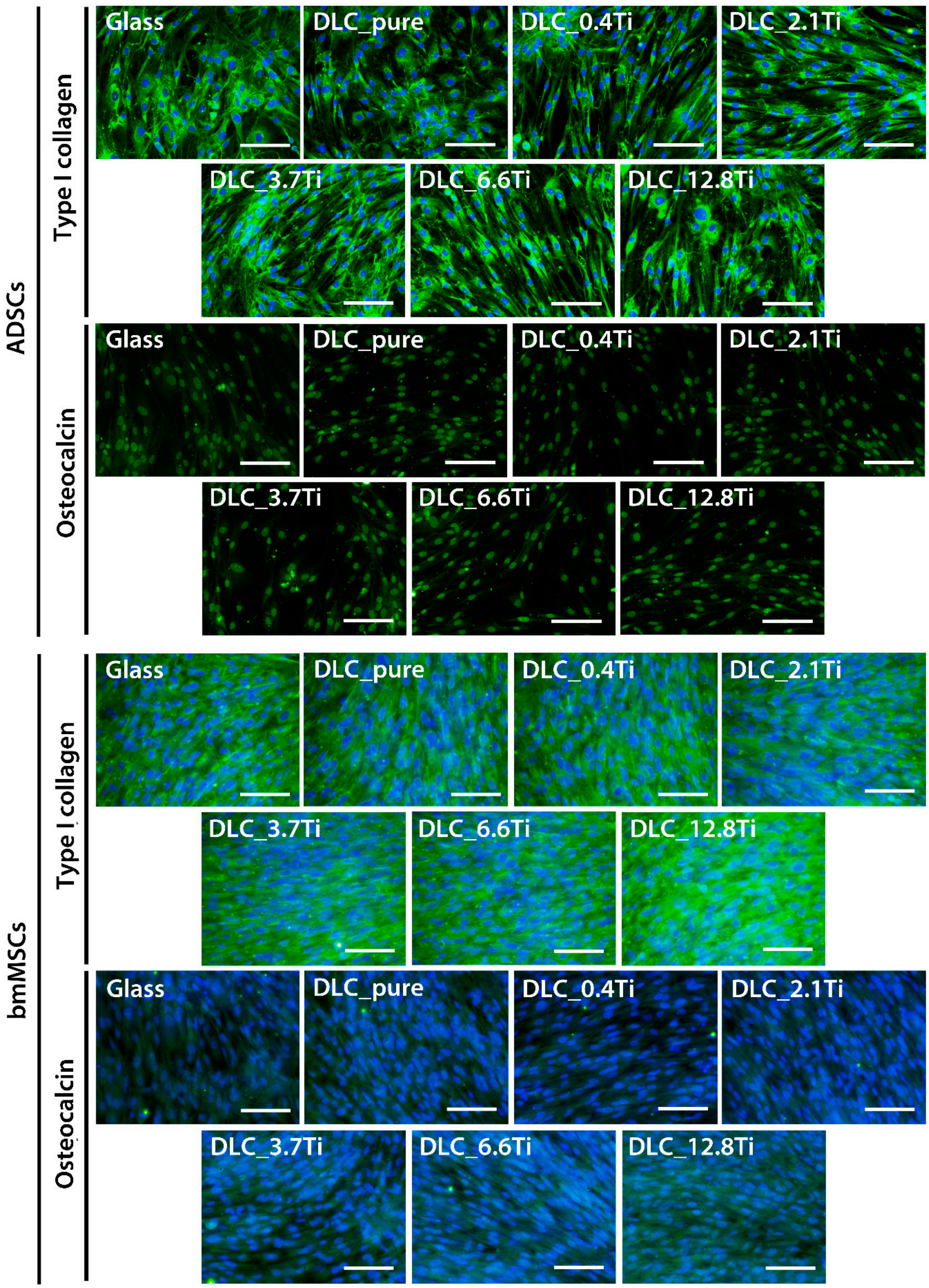

Figure S2. The immunofluorescence staining of type I collagen and osteocalcin (green colour) in ADSCs and bmMSCs on glass, on pure DLC, and on DLC doped with Ti (0.4, 2.1, 3.7, 6.6, 12.8 at.% of Ti) on day 7. Cell nuclei are counterstained with Hoechst 33258 (blue). Representative images were selected. Olympus IX71 microscope, IX71 digital camera, objective ×20, scale bar = 100 μm.

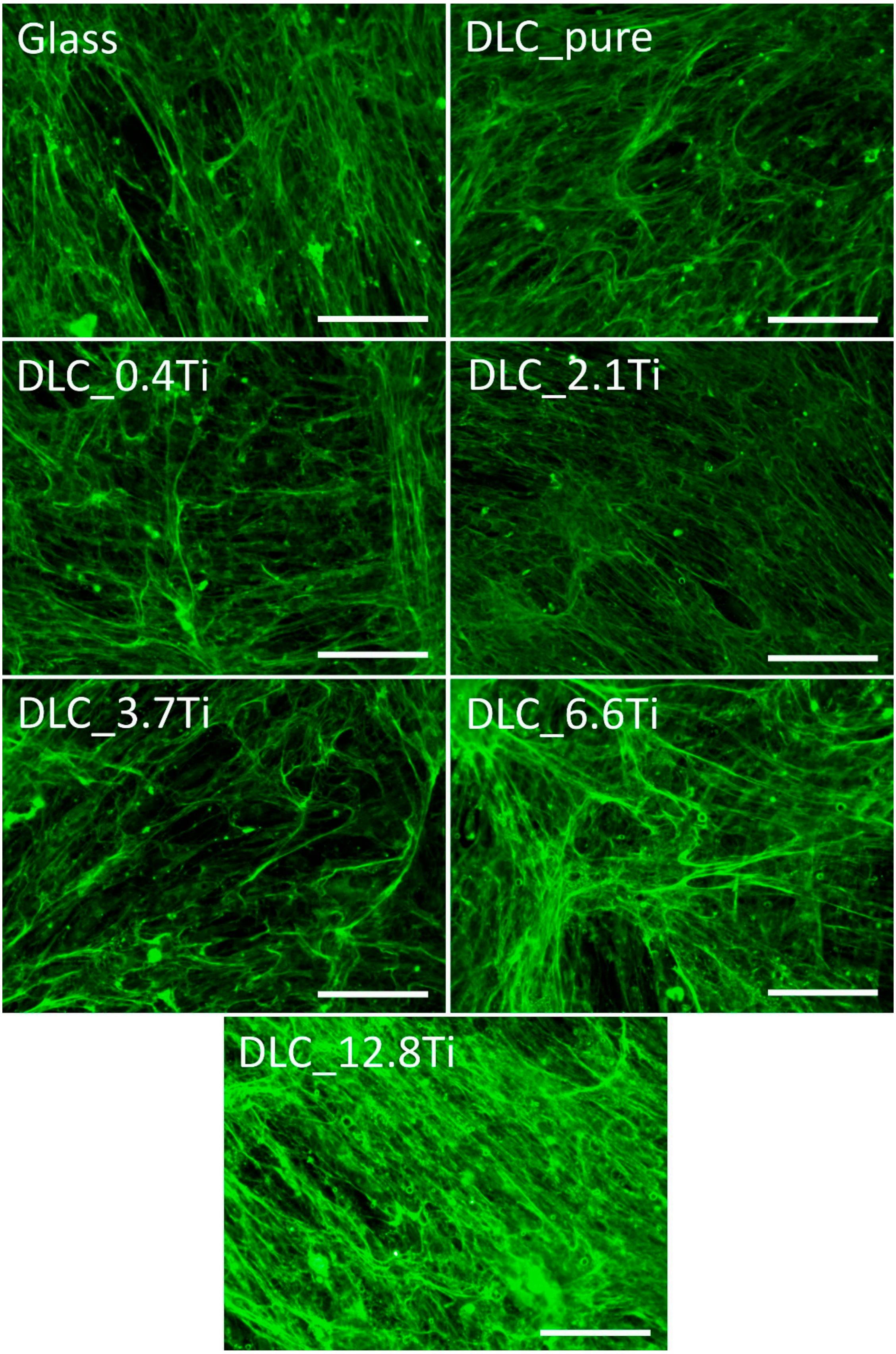

Figure S3. The immunofluorescence staining of type I collagen in ADSCs on glass, on pure DLC, and on DLC doped with Ti (0.4, 2.1, 3.7, 6.6, 12.8 at.% of Ti) on day 14. Representative images were selected. Olympus IX71 microscope, IX71 digital camera, objective ×20, scale bar = 100 μm.

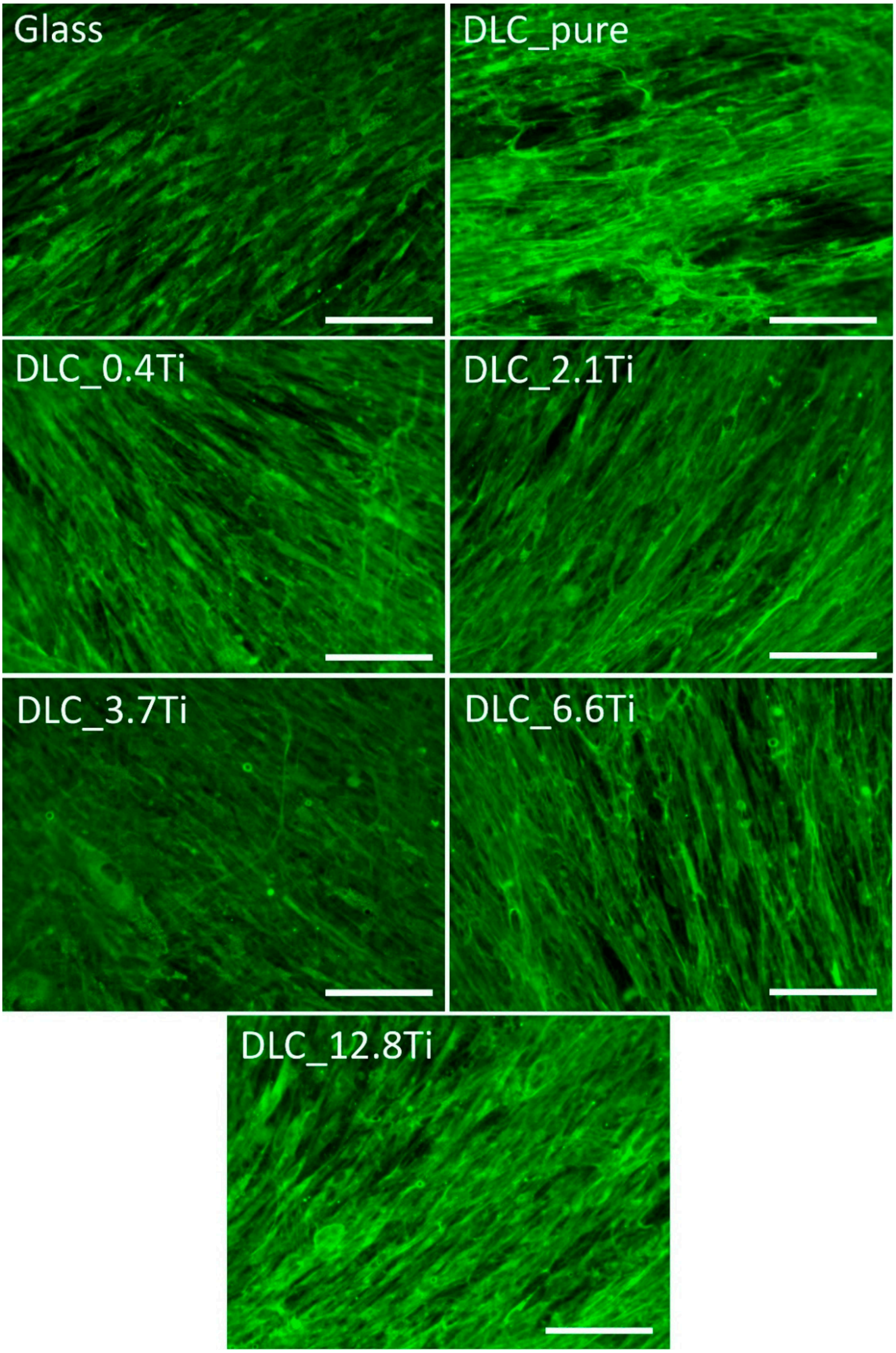

Figure S4. The immunofluorescence staining of type I collagen in ADSCs on glass, on pure DLC, and on DLC doped with Ti (0.4, 2.1, 3.7, 6.6, 12.8 at.% of Ti) on day 21. Representative images were selected. Olympus IX71 microscope, IX71 digital camera, objective ×20, scale bar = 100 μm.

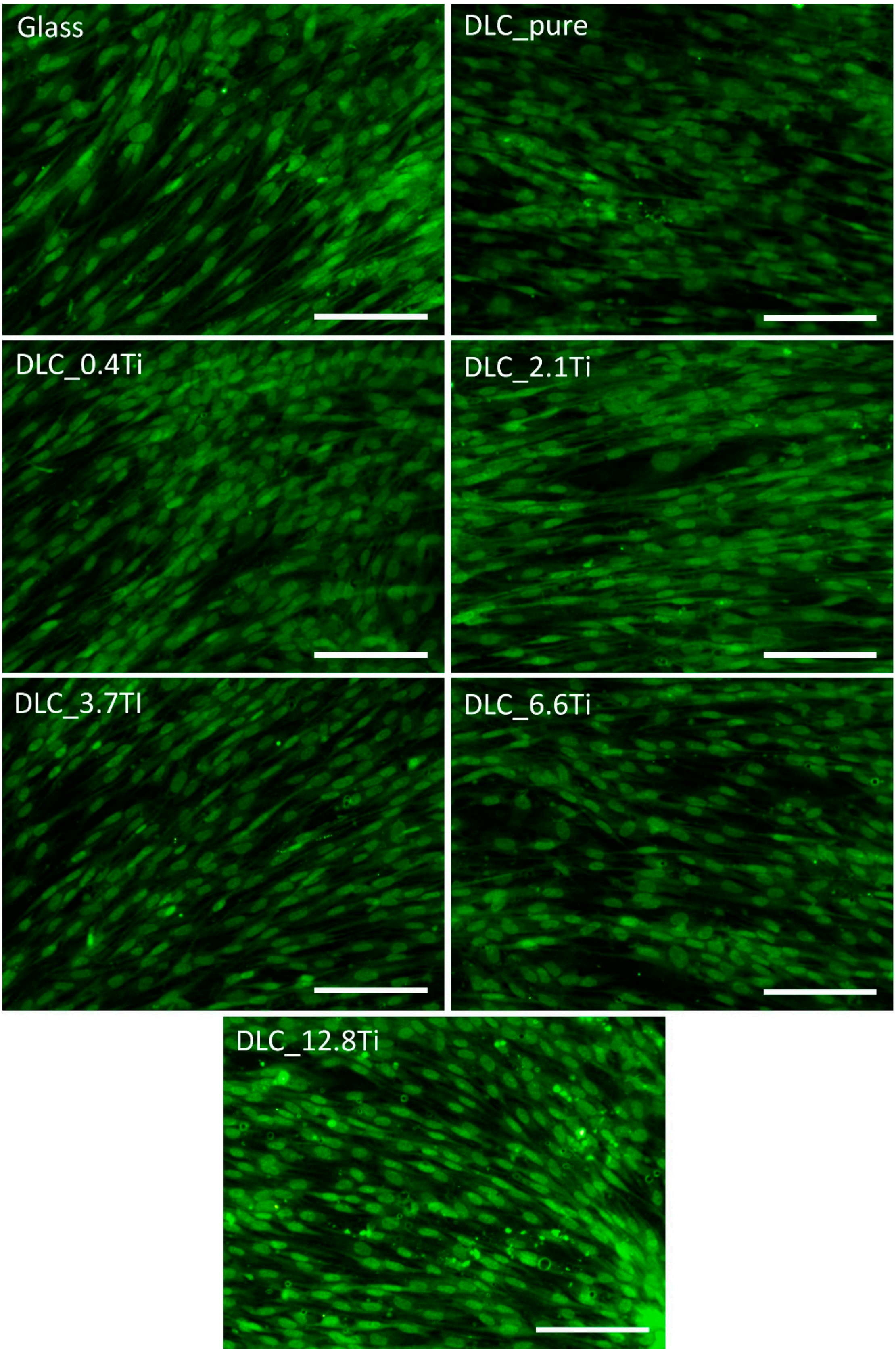

Figure S5. The immunofluorescence staining of osteocalcin in ADSCs on glass, on pure DLC, and on DLC doped with Ti (0.4, 2.1, 3.7, 6.6, 12.8 at.% of Ti) on day 14. Representative images were selected. Olympus IX71 microscope, IX71 digital camera, objective ×20, scale bar = 100 μm.

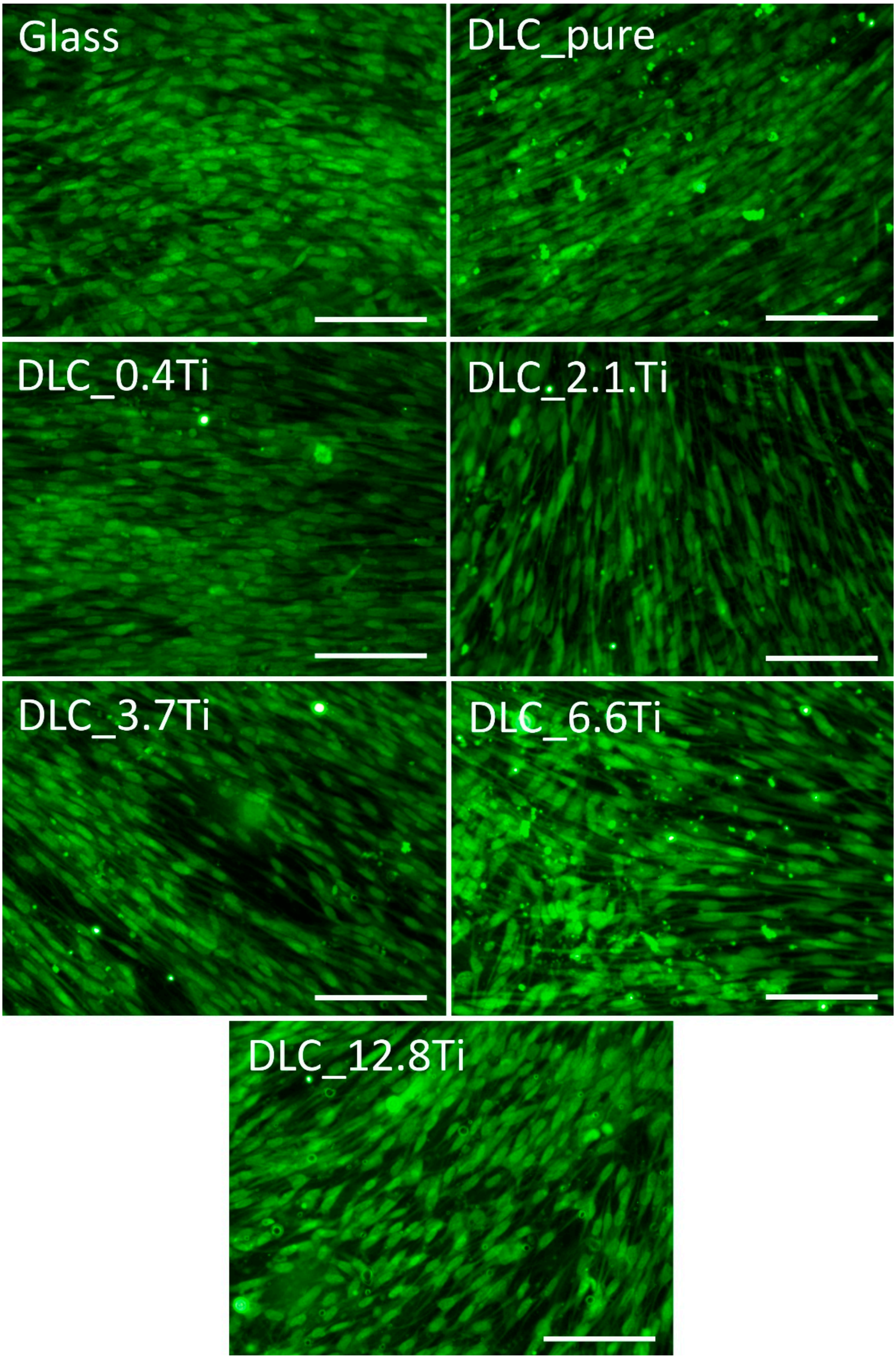

Figure S6. The immunofluorescence staining of osteocalcin in ADSCs on glass, on pure DLC, and on DLC doped with Ti (0.4, 2.1, 3.7, 6.6, 12.8 at.% of Ti) on day 21. Representative images were selected. Olympus IX71 microscope, IX71 digital camera, objective ×20, scale bar 100 µm.

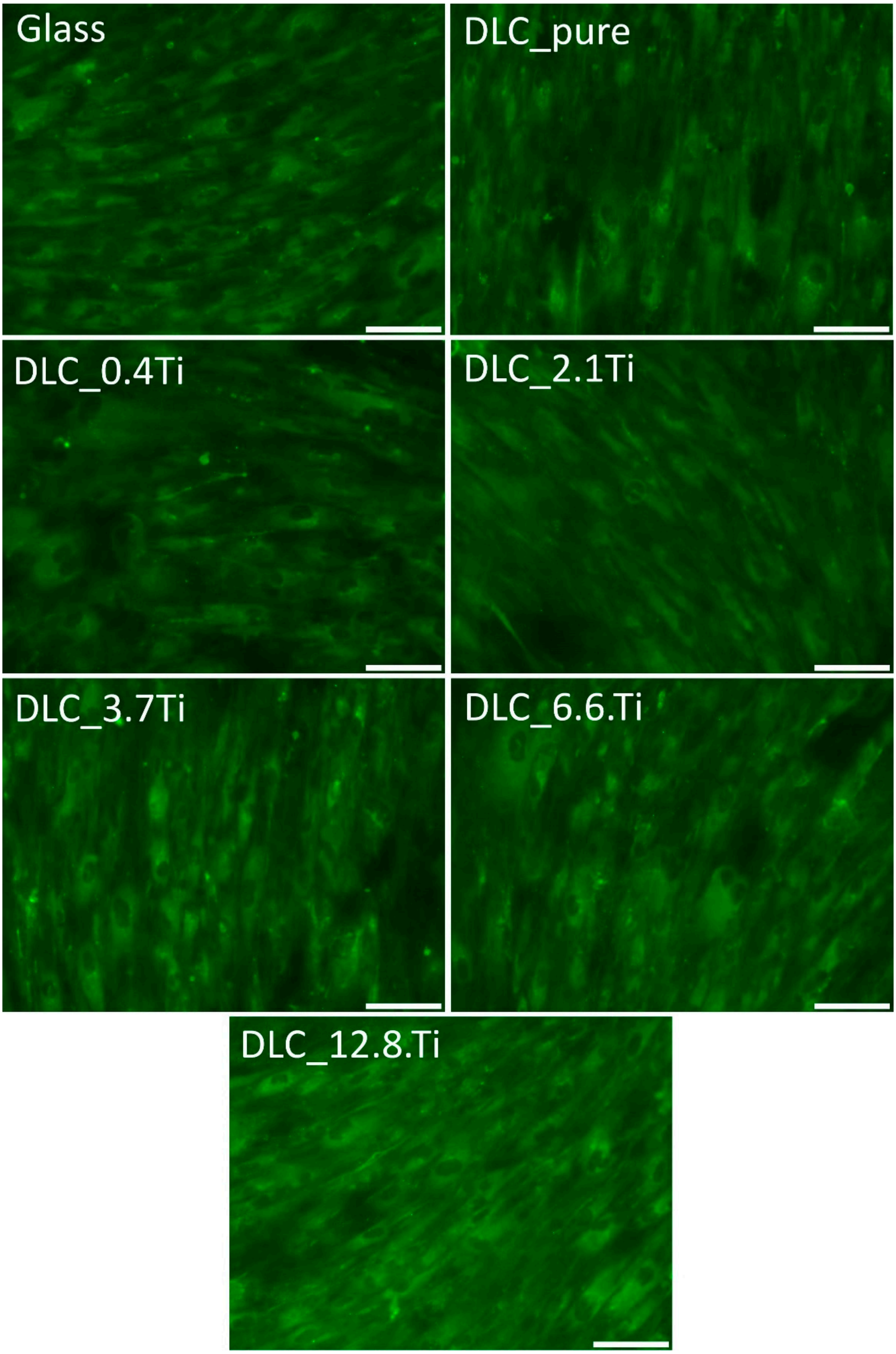

Figure S7. The immunofluorescence staining of type I collagen in bmMSCs on glass, on pure DLC, and on DLC doped with Ti (0.4, 2.1, 3.7, 6.6, 12.8 at.% of Ti) on day 14. Representative images were selected. Olympus IX71 microscope, IX71 digital camera, objective ×20, scale bar = 100 μm.

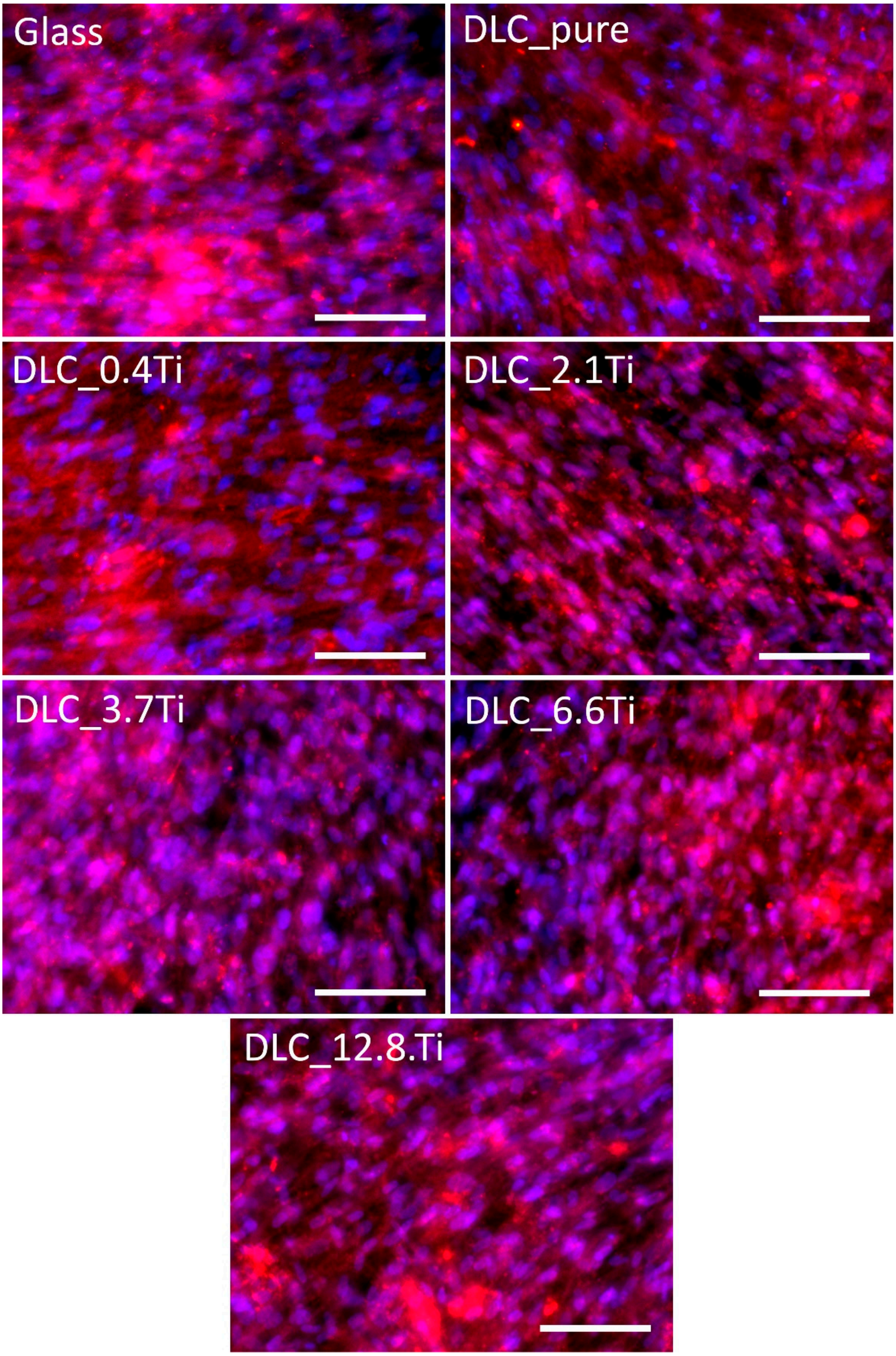

Figure S8. The immunofluorescence staining of type I collagen in bmMSCs on glass, on pure DLC, and on DLC doped with Ti (0.4, 2.1, 3.7, 6.6, 12.8 at.% of Ti) on day 21. Representative images were selected. Olympus IX71 microscope, IX71 digital camera, objective ×20, scale bar = 100 μm.

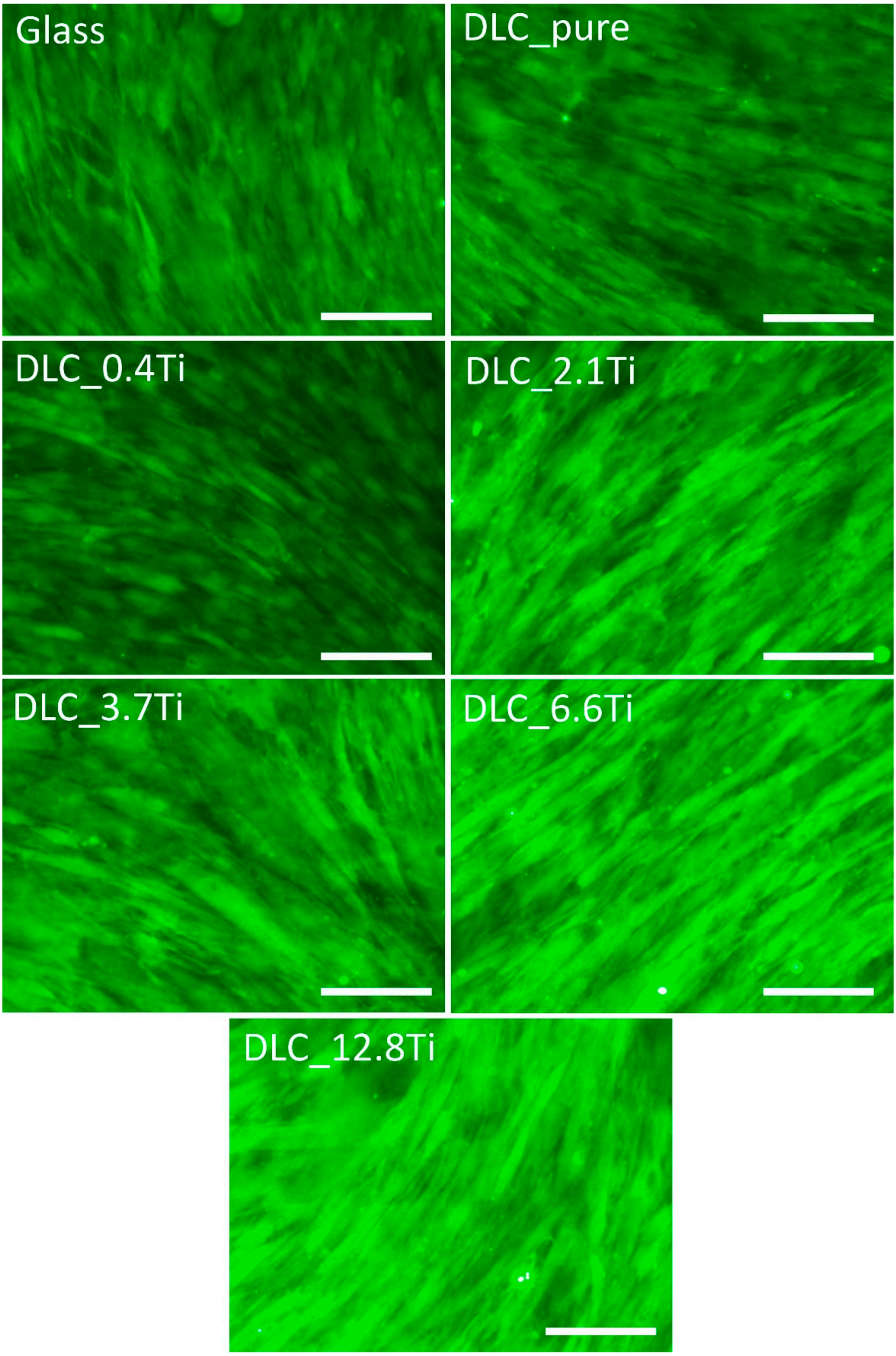

Figure S9. The immunofluorescence staining of osteocalcin in bmMSCs on glass, on pure DLC, and on DLC doped with Ti (0.4, 2.1, 3.7, 6.6, 12.8 at.% of Ti) on day 14. Representative images were selected. Olympus IX71 microscope, IX71 digital camera, objective ×20, scale bar 100 μm.

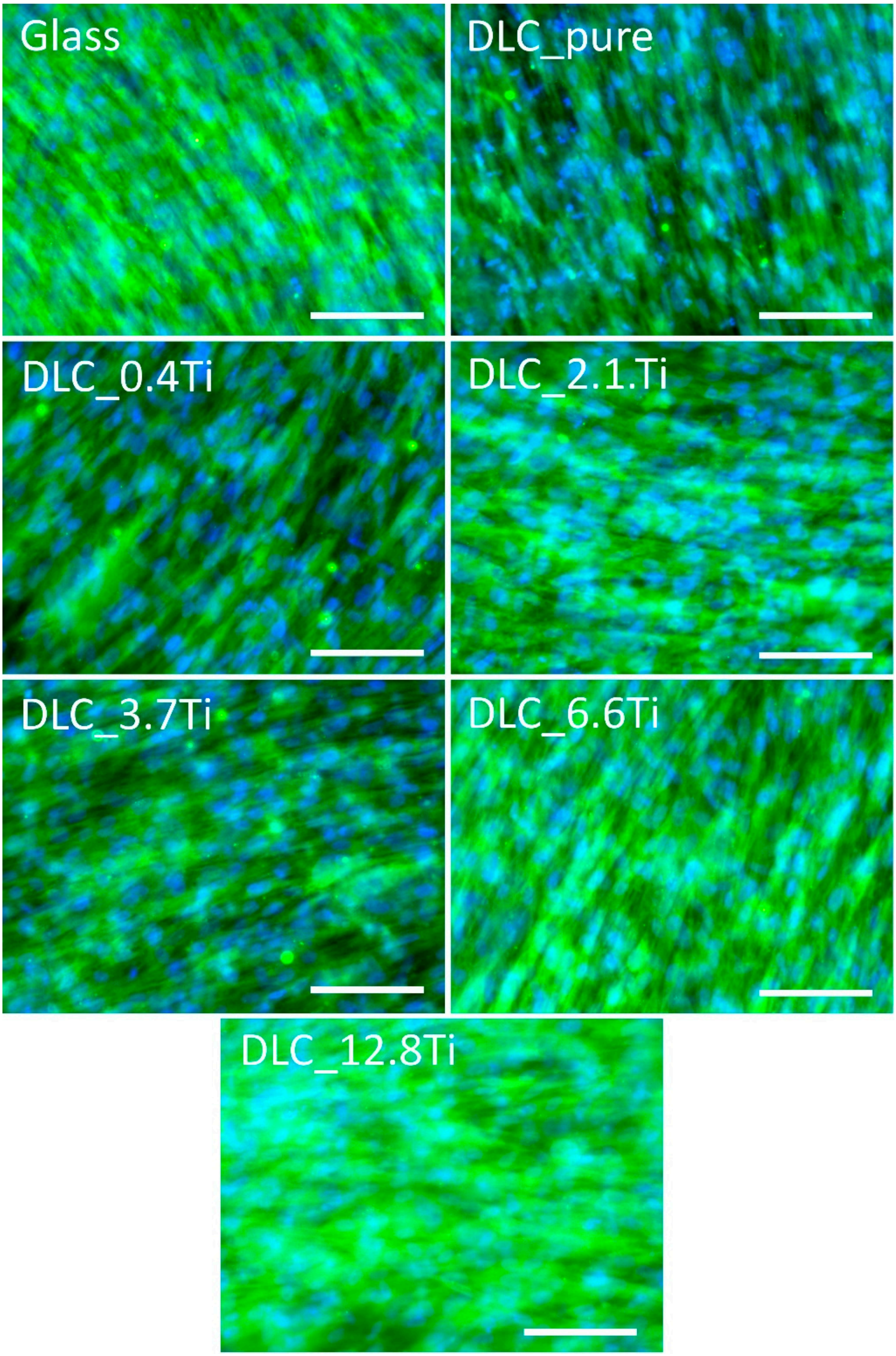

Figure S10. The immunofluorescence staining of osteocalcin in bmMSCs on glass, on pure DLC, and on DLC doped with Ti (0.4, 2.1, 3.7, 6.6, 12.8 at.% of Ti) on day 21. Cell nuclei are counterstained with Hoechst 33258 (blue). Representative images were selected. Olympus IX71 microscope, IX71 digital camera, objective ×20, scale bar = 100 μm.

Table S1. XPS take-off angle 0° to the perpendicular of the sample surface.

Sample	Atomic concentration [at.%]								
	C	O	N	Ti	Na	Ca	Si	Sn	Mg
Glass	30.95	43.22	-	-	2.66	1.46	18.54	0.26	2.90
DLC_pure	94.13	4.69	1.18	-	-	-	-	-	-
DLC_0.4Ti	93.45	4.68	0.97	0.89	-	-	-	-	-
DLC_2.1Ti	90.42	6.15	1.59	1.84	-	-	-	-	-
DLC_3.7Ti	91.00	5.85	0.31	2.85	-	-	-	-	-
DLC_6.6Ti	82.67	9.71	1.19	6.43	-	-	-	-	-
DLC_12.8Ti	68.41	21.46	0.68	9.45	-	-	-	-	-

Table S2. XPS take-off angle 81° to the perpendicular of the sample surface.

Sample	Atomic concentration [at.%]								
	C	O	N	Ti	Na	Ca	Si	Sn	Mg
Glass	41.28	45.47	-	-	1.03	0.85	9.48	0.11	1.78
DLC_pure	84.47	10.70	4.83	-	-	-	-	-	-
DLC_0.4Ti	89.29	8.92	1.79	-	-	-	-	-	-
DLC_2.1Ti	85.96	12.03	1.18	0.83	-	-	-	-	-
DLC_3.7Ti	87.28	9.57	1.63	1.52	-	-	-	-	-
DLC_6.6Ti	84.92	6.38	6.16	2.54	-	-	-	-	-
DLC_12.8Ti	86.90	9.40	-	3.71	-	-	-	-	-

Table S3. Detailed EDS analysis.

Sample	Weight percentage [wt.%]								
	C	O	Ti	Na	Mg	Al	Si	K	Ca
Glass	3.90	28.12		9.68	3.00	0.46	47.42	0.39	7.04
DLC_pure	33.24	14.35		6.39	2.23	0.35	37.60	0.26	5.58
DLC_0.4Ti	30.59	14.76	0.99	6.61	2.27	0.39	38.46	0.26	5.66
DLC_2.1Ti	27.67	16.09	3.31	7.36	2.35	0.39	36.99	0.29	5.56
DLC_3.7Ti	23.13	16.01	4.69	7.67	2.39	0.55	38.70	0.34	6.52
DLC_6.6Ti	16.81	18.03	7.81	7.13	2.48	0.39	40.84	0.32	6.18
DLC_12.8Ti	14.18	14.10	16.18	6.67	2.30	0.38	39.85	0.35	5.99
